# Exploratory Analysis of the Copy Number Alterations in Glioblastoma Multiforme

**DOI:** 10.1371/journal.pone.0004076

**Published:** 2008-12-30

**Authors:** Pablo Freire, Marco Vilela, Helena Deus, Yong-Wan Kim, Dimpy Koul, Howard Colman, Kenneth D. Aldape, Oliver Bogler, W. K. Alfred Yung, Kevin Coombes, Gordon B. Mills, Ana T. Vasconcelos, Jonas S. Almeida

**Affiliations:** 1 Department of Bioinformatics and Computational Biology, The University of Texas M. D. Anderson Cancer Center, Houston, Texas United States of America; 2 Department of Neuro-Oncology, The University of Texas M. D. Anderson Cancer Center, Houston, Texas United States of America; 3 Department of Pathology, The University of Texas M. D. Anderson Cancer Center, Houston, Texas United States of America; 4 Department of Neurosurgery, The University of Texas M. D. Anderson Cancer Center, Houston, Texas United States of America; 5 Department of Systems Biology, The University of Texas M. D. Anderson Cancer Center, Houston, Texas, United States of America; 6 Laboratório Nacional de Computação Científica, Laboratório de Bioinformática, Petrópolis, Rio de Janeiro, Brasil; 7 Instituto de Tecnologia Química e Biológica, Universidade Nova de Lisboa, Lisboa, Portugal; Columbia University, United States of America

## Abstract

**Background:**

The Cancer Genome Atlas project (TCGA) has initiated the analysis of multiple samples of a variety of tumor types, starting with glioblastoma multiforme. The analytical methods encompass genomic and transcriptomic information, as well as demographic and clinical data about the sample donors. The data create the opportunity for a systematic screening of the components of the molecular machinery for features that may be associated with tumor formation. The wealth of existing mechanistic information about cancer cell biology provides a natural reference for the exploratory exercise.

**Methodology/Principal Findings:**

Glioblastoma multiforme DNA copy number data was generated by The Cancer Genome Atlas project for 167 patients using 227 aCGH experiments, and was analyzed to build a catalog of aberrant regions. Genome screening was performed using an information theory approach in order to quantify aberration as a deviation from a centrality without the bias of untested assumptions about its parametric nature. A novel Cancer Genome Browser software application was developed and is made public to provide a user-friendly graphical interface in which the reported results can be reproduced. The application source code and stand alone executable are available at http://code.google.com/p/cancergenome and http://bioinformaticstation.org, respectively.

**Conclusions/Significance:**

The most important known copy number alterations for glioblastoma were correctly recovered using entropy as a measure of aberration. Additional alterations were identified in different pathways, such as cell proliferation, cell junctions and neural development. Moreover, novel candidates for oncogenes and tumor suppressors were also detected. A detailed map of aberrant regions is provided.

## Introduction

Copy number alterations (CNAs) are known to be among the triggers of tumor formation [1], [2]. Furthermore, tumor progression is associated with further variation in the copy number [3]. Although the association between chromosomal aberration and cancer has been known for a long time [4], recent advances in array-based techniques allowed a more refined description of the genomic structure, thus yielding a better characterization of copy number events.

Beginning with BAC and cDNA arrays [5], [6], the resolution of the array techniques increased to the current sensitivity level capable of detecting events with a size of thousands of base pairs [7], [8]. As a consequence of ongoing methodological advancement, a growing number of new oncogenes and tumor suppressors have been identified [9].

During cancer progression, tumor cells undergo several genomic changes. Mutations that enhance tumor progression are most likely to be positive selected in the neoplasm environment, and the cells that carry these mutations tend to be dominant in the tumor [10]. Due to the nature of CNAs, some mutations might carry genes that confer selective advantage, along with genes that do not. This creates a mutation background that obfuscates the localization of the major players of cancer [11]. Furthermore, tumor progression may take various routes as it occurs in the context of an individual genome and individual cell physiology. To track this variability, the analysis of several patients can be used to identify the recurrent regions of aberration (RRA).

Currently, most of the available mathematical tools for analyzing copy number data deal with segmentation methods and breakpoint detection [12], [13]. These techniques are used to define discrete regions in the genome that have the same copy number, analyzing each sample individually. However, few studies have addressed the detection of RRA, which are common amplification or deletions that occur in the same locus in a group of samples. One common approach to detecting RRA is to define arbitrary thresholds to identify amplifications and deletions [14], using the frequency of events as a measure of abnormality for a given region in the genome. However, the signal from each experimental platform may differ in variance, which implies that a different threshold may be needed for each new analysis. Furthermore, tumor samples typically contain normal cells that contaminate the tumor DNA, thus altering the amplitude of copy number aberrations. Using absolute copy number values as thresholds to segment CNAs ignores both confounding effects. A number of other studies report more sophisticated methodologies for RRA detection, but they still rely on arbitrary calling for amplifications and deletions [11], [15]–[17].

In this study, we propose a new method for identifying RRA based on the information content of each probe position. The main goal is to provide an approach that detects aberrant regions while making minimal assumptions about their nature, scale or prevalence.

Another aim of this study is to provide an exploratory framework for analyzing the data from glioblastoma multiforme (GBM) patients generated by The Cancer Genome Atlas project (TCGA;[18]). Despite the recent advances in the molecular pathology of GBM, the underlying mechanism of the origin and invasiveness of malignant glioma remains obscure [19].

As often noted in quality analysis surveys [20], data analysis results without dissemination of the applications that generated them are of unknown reproducibility. Therefore, an accompanying graphical tool is included to provide analysis of the copy number results as they are made available by the TCGA project and, more specifically, to allow the reproduction of the results reported here.

## Results and Discussion

### Exploring the TCGA data

In order to analyze the data generated by the TCGA project, a new graphical tool was developed, the Cancer Genome Browser (CGB), which is freely available at http://code.google.com/p/cancergenome/. The rationale for this tool is to provide a client application that can be used for the visualization and data processing reported here. In the tool, the input data is directly accessed from a semantic database [21] that provides the TCGA raw data in data structures designed to support the graphic representations reported here. The raw copy number data is stored alongside its preprocessed segmented values.

### Assessment of aberration

We present a new mathematical method that uses Shannon's entropy as a measure of genomic aberration. The entropy measures the deviation from the common state in a system. In the genomic context, the common state would be that all the samples have a copy number around 2. Any deviation from that state should be reflected in the entropy so that the more aberrant a region is, the lower the entropy.

**Figure 1:**
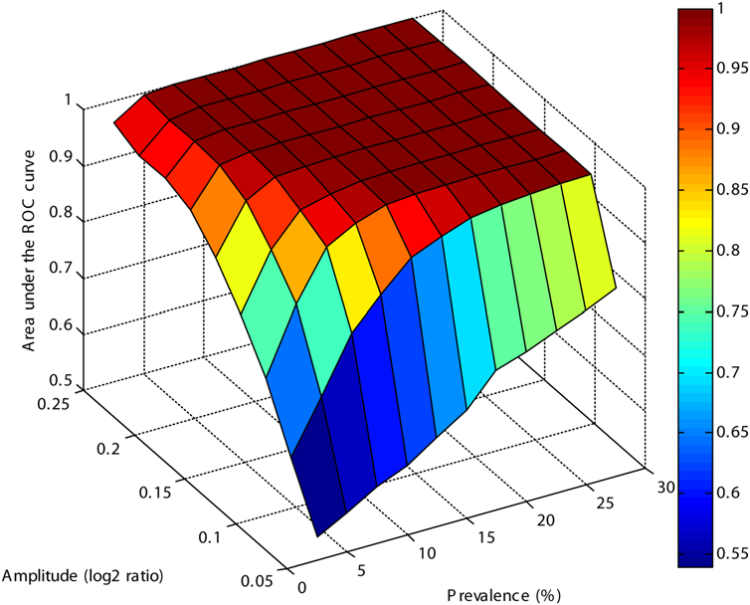
Evaluation of the entropy method performance by the area under the ROC curve from simulated datasets, accessing different amplitudes and prevalences of copy number aberrations. An area under the ROC curve of 1 means a perfect separation between mutated and normal regions, while a value of 0.5 means a random classification.

The detection of aberrant regions by the proposed procedure was first assessed using a simulation study (see Methods). Goodness of classification was determined using the area under the receiver operating characteristic curve, with 1 indicating perfect recognition of all alterations and 0.5 indicating random classification of the variation. Several simulated datasets were tested, encompassing different combinations of amplitudes and prevalences (the frequency of mutation in the population). In each one, a determined region of the genome had its copy number values added (amplified) by a certain amplitude value in a fraction of samples (controlled by the prevalence). The results were virtually the same when deletions were tested instead of amplifications (data not shown). The area under curve results are displayed in Figure 1, where it can be observed that, for amplitudes greater than 0.2, a perfect classification can be obtained if the prevalence is greater than 5%.

There are two main forms of CNA [11]: broad events, which can encompass several Mb or even the whole chromosome, and focal events, which are normally restricted to a few Mb. The search for new oncogenes and tumor suppressors in broad events can be extremely difficult due to the large number of genes within these regions. Therefore, we chose to analyze only the focal events and remove the influence of broad events in the entropy analysis. This was done by performing the analysis in each chromosome separately (thus nullifying the influence of whole chromosome aberrations) and applying baseline removal techniques to reduce the effects of other broad events in the entropy signal (see Methods).

### GBM analysis

A total of 169 RRA were found using the 167 tumor samples (Table S1), being the majority of these regions annotated as copy number variation (CNV) that occurs in normal samples. CNV in normal cells has recently been described as a relatively common occurrence in the human genome [22]. To separate the mutations related to tumor progression from the normal CNV, the results were screened to identify the regions in which more than 50% of the probes were annotated as normal CNV or were detected in low-entropy peaks in normal samples. Thirty-one regions passed this test and were the objects of further analysis (Table 1 and Figure 2). The chromosomes X and Y were not analyzed. The entropy analyses for each chromosome are available on Figures S1, S2, S3, S4, S5, S6, S7, S8, S9, S10, S11, S12, S13, S14, S15, S16, S17, S18, S19, S20, S21, S22.

**Figure 2:**
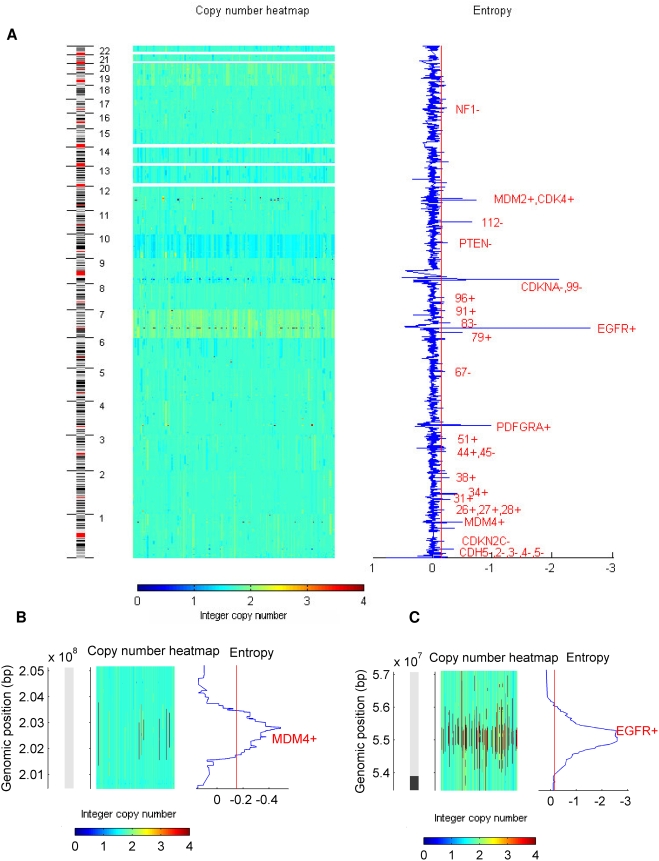
Entropy analysis for the GBM data entire genome (A), chromosome 1(B) and chromosome 7 (C). The segmented copy number values per sample are displayed as a heatmap on the left, with the tumor samples as columns; the entropy values are shown on the right. The gaps on the heatmap indicate genomic regions that lack coverage, such as the p arms of acrocentric chromosomes. The number in A corresponds to the region number in Table 1. Beside each region label, a plus sign indicates an amplification and a minus indicates a deletion. Note that a low-entropy region can be either a amplification or a deletion. The red line in the entropy plot shows the threshold for defining an aberrant region, which is the 0.05 quantile of the bootstrap distribution of the entropy. Peaks that are below the threshold but have no region assigned are normal CNV. The cytoband annotation was retrieved using the UCSC Table Browser.

**Table 1:** Amplifications and deletions in the GBM data.

Type	Region #	Known genes in GBM	Candidates	Ch	Start	End	Entropy	# of genes	Amplitude	Prevalence
**Amplification**	82	EGFR		7	54027966	55983910	−2.5865	7	0.000275	0.234739
	55	PDFGRA		4	53634367	57090231	−0.94003	22	0.000708	0.139728
	117	CDK4		12	56097914	56851736	−0.69274	26	0.001321	0.122698
	118	MDM2		12	67315903	67981156	−0.49955	7	0.000446	0.103178
	79			7	32978614	32991778	−0.48806	1	0.002777	0.101796
	22	MDM4		1	201942811	203355851	−0.48298	19	0.000311	0.084066
	34			2	113106316	113112980	−0.4263	0	0.000597	0.083832
	38			2	202864197	203906650	−0.26864	9	0.014994	0.038269
	51			3	181382003	181447344	−0.22919	0	0.000371	0.071856
	44			3	106776838	106776898	−0.21799	1	0.003143	0.047904
	91			7	152135252	152147233	−0.20795	1	0.005562	0.05988
	96		SGK3*	8	67783070	68102356	−0.18707	3	0.017116	0.041916
	27		NCOA1*	2	24616100	24712348	−0.18293	1	0.008564	0.053892
	26			2	24426149	24551934	−0.16724	1	0.008564	0.05988
	31		ATOH8*	2	85806436	85899917	−0.15732	1	0.012083	0.047904
	28			2	24819254	24866786	−0.15088	2	0.008564	0.047904
**Deletion**	100	CDKN2A		9	20336123	24769734	−1.89499	25	0.001192	0.258973
	112			11	70513145	70559392	−0.67419	0	0.018841	0.052181
	3	CHD5	AJAP1	1	4028404	6333694	−0.36827	10	0.004095	0.137835
	9	CDKN2C		1	50961735	51283220	−0.33261	2	0.000366	0.066068
	99			9	20240063	20280240	−0.31992	0	0.002421	0.147705
	83			7	86779323	86785351	−0.26366	0	0.000305	0.065868
	106	PTEN		10	89545841	89680005	−0.24913	3	0.000401	0.06373
	5		TNFRSF9	1	7889543	7926197	−0.23605	1	0.000965	0.155689
	2			1	3928417	3978407	−0.21835	0	0.006145	0.113772
	4		CAMTA1*	1	7728761	7742417	−0.21773	1	0.005078	0.149701
	146			17	28797037	29337463	−0.2158	1	0.01771	0.054943
	45		LSAMP	3	117520637	117525922	−0.2012	1	0.015102	0.05988
	145	NF1		17	26438606	26453055	−0.19121	1	0.004396	0.053892
	67			5	164370828	164522125	−0.19096	0	0.013057	0.05988
	147		ACCN1	17	29404279	29546036	−0.17745	1	0.021171	0.053892

Aberrant regions determined by the entropy method. These tables do not include regions for which more then half of the DNA probes were annotated as normal CNV. Candidate oncogenes / suppressors were selected by their potential association with cancer based on their molecular function or for being the only gene in the region. The genomic mapping was based on Build 18. The calling for amplifications and deletions was made by observing which amplitude measure, QQ0.025 (deletion) or 1-QQ0.975 (amplification), was closer to 0.

* Not described or validated as oncogene or tumor suppressor in previous studies.

Among those 31 regions, there are 10 genes known to modulate cell proliferation in GBM: EGFR, MDM2, MDM4, CDK4, PTEN, PDFGRA, CDKN2A, CDKN2C, NF1 and CHD5 [23], [24]. However, the total number of CNAs involved in cell proliferation is still unknown, and recent studies have added new genes to the pool of oncogenes and suppressors of GBM [11], [24], [25].

### Amplitude and prevalence of the CNA

The rationale of the entropy method allows a straightforward interplay between the prevalence and amplitude of CNAs. Some of the detected mutations, such as MDM4 and PTEN, have a low prevalence in the population but high copy number amplitude. Conversely, other regions, such as #51, have relatively low amplitude and a high prevalence.

Measures of the amplitude and prevalence directly on the log2 ratio copy number can lead to bias due to the whole chromosome variation and broad events. To avoid this problem, the amplification prevalence in an aberrant region was measured as the proportion of probes within the region, considering all samples, with a copy number above the 0.975 quantile of all copy number values for all samples at the same chromosome. For deletions, the proportion of values below the 0.025 quantile was used.

**Figure 3:**
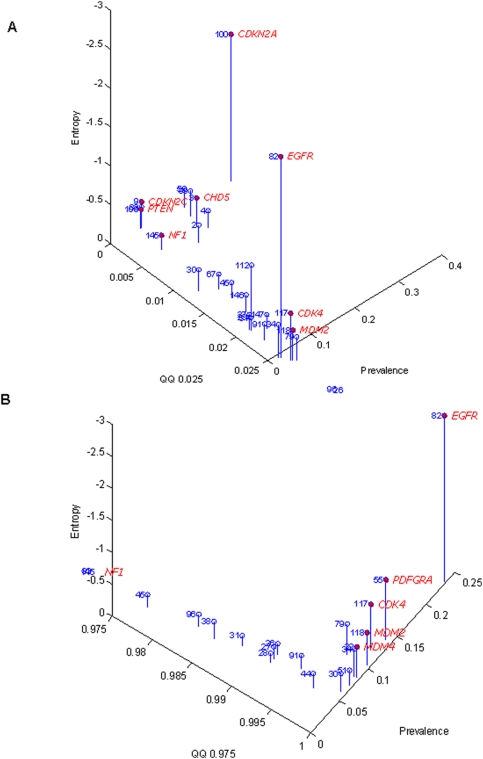
Interplay between the amplitude, prevalence and entropy in deletions (A) and amplifications (B). The prevalence measure adopted is the proportion of DNA probes on determined regions that have a log2 ratio above or below the 0.025 and 0.975 quantiles.

The amplitude of a given probe position was obtained in a similar way: considering all samples and all probes within the region, the 0.975 and 0.025 quantiles of the copy number of the aberrant region were obtained. Then the quantile of these values, in comparison with the copy number for all the probes in the same chromosome, was used as a measure of amplitude called QQ0.025 and QQ0.975. For instance, let q be the 0.975 quantile among all the copy number values from all samples within a determined aberrant region. The amplitude of amplification, QQ0.975, for that region will be the quantile relative to q when compared to all of the copy number values for the same chromosome. If the observed region was not aberrant, QQ0.975 would be around 0.975. Consequently, a QQ0.975 close to 1 indicates amplification, and a QQ0.025 close to 0 indicates deletion. To consider an aberration as an amplification or deletion alike, the one tailed area is considered (which is to say, the area at either end of the two tailed distribution) and the amplification amplitude is expressed as 1- QQ0.975. Interestingly, the known aberrations for GBM tend to have the most extreme values of amplitude and are therefore found at the end of the distribution tails with QQ0.025 and 1- QQ0.975 values close to 0 (Figure 3). The separation between amplification and deletion is done by observing each value, QQ0.025 or 1-QQ0.975, is close to 0.

### Genes within low-entropy regions

The recurrence of an aberration can be related to the influence of its genes in tumor progression [10]. However, some regions can contain other genes that may not be related to cancer. In [11], the genes that have influence on cancer are designated as “drivers” and the genes that are near the “drivers” but have no effect on tumor progression are designated as “passengers.” Most authors assign only one “driver” per region [7], [11], [14], but there may be other genes related to cancer within the same region. For instance, region # 100 in Table 1, which contains the known described tumor suppressor CDKN2A, also contains the gene ELAVL2, which is related to neuronal proliferation and differentiation [26]. A cluster of interferon genes is also present in this region and can influence tumor growth and progression [27]. Another example of a region with more than one “driver” is region #117. Besides the well-described oncogene CDK4, this region also contains glioma-associated gene 1, which has been described as affecting cell proliferation and differentiation [28]. However, the CNA that is most likely to have multiple “drivers” is the well-known deletion of 1p36 [25], [29]. This deletion is present in gliomas and neuroblastomas, but a single “driver” could not be defined [29] in previous studies. Together with the tumor suppressor CDH5, analysis of the low-entropy regions shows that the genes TNFRSF9, CAMTA1 and AJAP1 are among the candidates for tumor suppressors. A complete list of genes found in low-entropy regions is available in Table S2.

The gene CDKN2C, a well-known tumor suppressor [30], was found in region #9 (Table 1). Being an important player in oligodrendroglioma and medulloblastoma, the deletion of CDKN2C was recently described in GBM [17]. Moreover, a deletion of gene NF1, a gene associated with neurofibromatosis type 1 that appears to be a negative regulator of the Ras pathway [31], was also detected.

Among the candidates for suppressors in GBM listed in Table 1 are the genes LSAMP and ACCN1. The former has been described as a tumor suppressor in renal carcinoma [32], and the latter has been described as an inhibitor of glioma cell proliferation [33]. Some new candidates for oncogenes in GBM have also been found. The gene ATOH8 is a transcriptional regulator related to glial determination [34], but has never been described as an oncogene. Finally, non-annotated normal CNVs might be the cause of some low-entropy peaks, as in regions #91 and 112, which are located close to known CNV events.

Paralog regions may contribute to the pool of detected of aberrant regions. An example of the former is region #79, which is paralog to the region 55715461–55763010 on chromosome 7 [35],that lies within the EGFR amplified area, The Pearson's correlation between the copy number values of the two paralog regions is 0.79.

### Comparison with other methods

The literature reports two methods for the identification of RRA that were applied to the TCGA glioblastoma dataset by the TCGA Research Network [36]: GISTIC [11] and GTS [17]. GISTIC uses an arbitrary threshold to define deletions and amplifications and calculates the q-value [37], which is an upper bound to the false discovery rate, as a measure of aberration. GTS searches for RRA using a statistic that considers the number of genes in aberrant area and their copy number value. It also uses arbitrary thresholds to define aberrant regions.

Despite the differences in the methods, the main mutations in GBM (EGFR, CDKN2A, CDKN2C, PDGFRA, PTEN, CDK4, MDM2 and MDM4) were correctly recovered by the entropy method and the TCGA Research Network analysis, which used a combination of methods that included GISTIC and GTS. Confirming the simulation results, the entropy analysis was insensitive to mutations with low prevalence (<4–5%). Some known oncogenes and suppressors in GBM were not detected by the entropy method, but were correctly identified by the combined GISTIC and GTS analysis (prevalence in parentheses): MET (3%), CDK6 (1%), TP53 (1%), CCND2 (2%) and PIK3CA (2%). The tumor suppressor RB1 was not detected by entropy because it is located in the peak of a broad deletion event and is obfuscated by the baseline removal. However, low prevalence mutations will always represent a challenge for statistical methods that consider the whole population in the analysis. With arbitrary thresholds for amplification and deletion of 1.5 and −1.5, respectively (log2 scale), some mutations were not detected by any method, such as CCNE1 and CCND3 (genes with a prevalence less than 1%).

The absence of any unique pattern on the GBM genotype and the influence of low prevalence mutations suggest that a better description of the copy number data can be achieved if individual characteristics of each sample is considered instead of a summary for the whole population. In that context, the entropy method should be used as an initial scan of the copy number data due to its speed, in the order of seconds, robustness and lack of parameter calibration. Future versions of the CGB tool will include GTS and GISTIC, thus allowing the integration of different methods of RRA detection with heatmap visualization and data exporting.

### Tumor vs. normal samples

**Figure 4:**
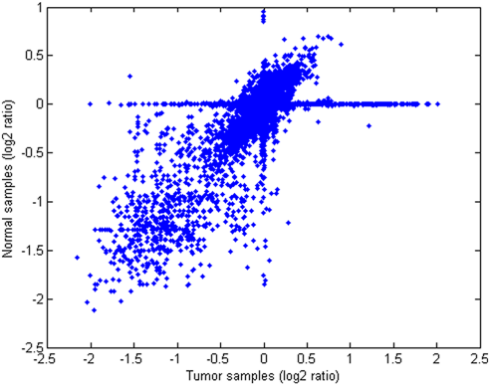
Log2 ratio of copy number of the 58 tumor-normal sample pairs. The values in the diagonal line correspond to variations similarly observed in tumor and normal tissue. The values in the horizontal line correspond to amplifications (right) and deletions (left) observed only in the tumor samples.

In this study, normal samples were used for identifying germline CNV (see Metods). However, the comparison of the paired normal and tumor samples reveals mutations that are only present in the normal samples, which contradicts the common assumption that blood samples contain only germline CNV (Figure 4). A detailed analysis of these mutations indicates that most of them are artifacts of the segmentation procedure, which creates very small segments in the normal samples that are not present in the tumor samples. Also, the samples from patient TCGA-06-0178 appears to be mislabeled; while the tumor sample has almost no mutation, the blood sample contains several CNAs, including the oncogene CDK4.

In a comparison between the low-entropy regions on the normal samples (Table S3) and the regions annotated in the CNV databases (described in Methods), 62% of the DNA probes of the low-entropy regions in normal samples are also located in known CNV regions. Moreover, some of the low-entropy regions are located close to a CNV, and it might be reasonable to assume that they are part of the CNV, once it is difficult to achieve a precise definition of the boundaries of a CNV with array techniques [38]. This observation suggests incompleteness of the current databases for normal CNV. Indeed, some of the low-entropy regions in normal samples (e.g. region 85219839–85227131 on chromosome 12) were later confirmed by sequencing to be CNVs [38]. Experimental artifacts may also be underlying reason for the low-entropy regions in normal samples.

### Conclusion

This study presents a new method for detecting RRA that uses low entropy as an indicator. A stand-alone graphic application is provided for the exploration of the TCGA data and the replication of the detection of low-entropy regions presented here.

From a dataset of 167 GBM samples from the TCGA project, 31 aberrant regions were found, including 10 known CNAs in GBM, namely the genes EGFR, MDM2, MDM4, CDK4, PDGFRA, PTEN, CDKN2A, CDKN2C, NF1 and CHD5. Also, candidates that were never described as being major players in cancer, such as the glial differentiation gene ATOH8 and the transcription factor NCOA1, were detected in aberrant regions. The unusual level of enrichment of the list of candidate oncogenes and tumor suppressors lends considerable expectation to those few regions for which neither variability nor association with tumor formation could be found.

The analysis of the entropy in the blood (non-tumor) samples showed that only 62% of the aberrant regions were previously annotated as normal CNV regions. The expansion of the CNV databases may refine the separation between normal CNV and copy number aberrations that have influence on cancer.

## Methods

### Source data

A total of 227 normalized array comparative genomic hybridization (aCGH) results for GBM patients were retrieved from the TCGA data portal (http://tcga-data.nci.nih.gov/). The aCGH experiments were performed by the Memorial Sloan-Kettering Cancer Center using the Agilent Human Genome CGH Microarray 244A (Agilent Technologies, Inc., Santa Clara, California) platform. From the 227 samples (Table S4), 167 were tumor samples and 60 were blood samples. When there was more than one sample of the same tissue for a patient, one sample was randomly selected (see Supplementary Material for a sample list). Of the 167 tumor samples, 58 had a paired blood sample from the same patient.

The normalized copy number data obtained from the 227 samples were mapped into the human genome using the Build 18 (NCBI 36) assembly with an annotation file provided by the manufacturer (http://www.chem.agilent.com/). The array normalization procedure was performed by Memorial Sloan Kettering Cancer Center with their in–house algorithm that corrects for CG contents bias (see TCGA Data portal; http://tcga.cancer.gov/dataportal). The copy number data was filtered using the Circular Binary Segmentation (CBS) algorithm as implemented in the R package DNAcopy with the default parameter settings [12].

### Data analysis method

The detection of aberrations was pursued here as that of an unqualified deviation. As a consequence of this critical concern with untested null models, the choice of method must satisfy two concerns about possible bias. First, it must make no assumptions about a reference non-deviant signal. Secondly, it must make no assumptions about the shape of the variation. These non-parametric requirements are satisfied by approaches that use the density of observed measures to assess the information content of the signal. The individual signal is thus assessed by the probability, *p*, of the deviation in the context of observed signals. Shannon's entropy (Eq. 1) was calculated for each of the DNA probe positions, i = 1,…,n.
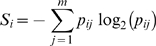
(1)


The probability, *p_ij_*, for each copy number value was determined as the fraction of the kernel density, K, observed in all samples, *j = 1,…m*, at that position (Eq. 2).
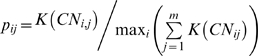
(2)


The Parzen window method [39] with the Gaussian kernel function was used to approximate the probability density value *K* of the log2 ratio of copy numbers observed at that position, *CN_i,j_*. This technique considers that every element in the population is a center of a Gaussian curve, and that the probability density value for a given point is the sum of all Gaussian values at that point. The calculation of the kernel density for all DNA probes would have required a large amount of computational effort. Therefore, the kernel density was sampled in 100 equally-distributed points, KS, ranging from the minimum to the maximum value of the copy number log2 ratio (Eq. 3). The probability density value, *K(CN_ij_)*, was then obtained by interpolation with the vector KS. The parameter *σ*, relative to the bandwidth of the kernel, was defined as the standard deviation of the raw data inside each segment summarized for all segments in all samples. Methods for bandwidth estimation that were designed for Gaussian populations yielded a bandwidth parameter that was too short, which resulted in several peaks in the probability density distribution (data not shown). Our bandwidth selection criteria resulted in a unimodal probability density centered at 0. Since most of the important CNAs have high amplitudes, and consequently low probably densities, the detection of aberrant regions is relatively insensitive to large bandwidth parameters.
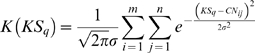
(3)


The amount of information associated with an “aberration” is inversely proportional to the entropy S. If a determined region is recurrently amplified or deleted, it should have a higher information content, and thus a lower entropy, when compared to the overall distribution of the entropy.

The implementation of this three step procedure is detailed using Matlab's m-code :

[n,m] = size(X);

1. Generate reference distribution
  [f,xi] = ksdensity(X(:), bandwidth);
  and replace each value by its density (Eq. 3)
  P = X;P(:) = interp1(xi,f,X(:));
2. Calculate the actual probability now as the proportion of row density (Eq. 2)
  P = P./repmat(max(sum(P,2)),1,m);
3. Calculate Shannon's entropy (Eq. 1)
  H = −sum(P.*log2(P),2);

Although applied to aCGH experiments in this work, the entropy method is suitable for any array-based copy number platform.

### Detecting the regions of interest

As discussed in [11], there are two main forms of CNAs in tumor cells: broad events, which can contain several Mb of nucleotides and encompass numerous genes; and focal events, which are much more localized. Focal events inside broad events represent a challenge for methods that are based on thresholds for the binary calling of amplifications and deletions, once the entire broad region can be considered significant, hence hidden the focal events. However, some methods for RRA detection, while relying on arbitrary thresholds, use the amplitude to separate these nested focal events [11], [17].

Even though broad events can be prevalent in the cancer genome [14], their applicability for finding new oncogenes or tumor suppressors is limited due to the large number of genes present in those regions. Thus, in this paper the detection of RRA was limited to the focal events. To remove the influence of entire chromosome amplification or deletion, the kernel density was calculated individually for each chromosome. Moreover, to diminish the effects of broad events on the entropy, the baseline of the entropy signal was removed using a Whitaker filter [40] (smoothing). For each probe position, the value of the entropy was determined as follows: Final entropy = Original entropy−Smoothed entropy. Therefore, only peaks in the entropy, which represent focal events, remained in the signal. Finally, a threshold for the entropy was obtained using the 0.05 quantile of the bootstrap distribution of the entropy. The regions that had a final entropy lower than the threshold were considered RRA. Regions represented by only one probe were not considered.

In the CGB tool, the baseline removal is given as an option to the user. Therefore, it is possible to deactivate this procedure in order to analyze broad events as well. Since the entropy method does not consider the size of the events, it is capable of detecting broad events such arm-size or even whole chromosome events. For whole chromosome events, the entropy should be measured in the whole genome instead of individually on each chromosome.

### Identifying normal CNV

CNV in normal cells has recently been described as a relatively common occurrence in the human genome [22]. To detect whether an RRA is a normal copy number variation or an aberrant alteration that promotes cell proliferation, the regions were compared to the entries of the Database of Genomic Variants (http://projects.tcag.ca/variation/; version 18v1; [22]) and the “Structural Variants” annotations in the UCSC Genome Browser [41]. Also, the entropy was calculated for the 60 normal samples using the same procedure described above. The low-entropy regions in normal samples were not used when analyzing the tumor dataset.

### Simulation of aberrant regions

One hundred simulations were performed to analyze the behavior of the entropy according to variations in the amplitude and the prevalence of CNAs. The length of each aberration was not changed since our method considers each position independently.

A set with 100 artificial patients was built using randomly sampled copy number values from the GBM data. The simulated CNA amplitude ranged from 0 to 0.4 (log2 ratio scale) with a prevalence from 0 to 25%. The area under the receiver operator characteristic curve (ROC) was used for performance evaluation in each simulated condition. The analysis of the simulation is described in the Results section.

## Supporting Information

Figure S1 Entropy analysis of chromosome 1, containing the copy number heatmap (on the right) and the entropy signal (left). The threshold for determining aberrant regions is displayed in the entropy plot as a red line, and it is defined by the quantile 0.05 of the bootstrap distribution of entropy. Only tumor samples are included. The assignments of the regions is the same on the Table 1 of the manuscript and peaks that don't have any regions assigned represent normal CNV or low-entropy regions in normal samples.
(0.26 MB TIF)

Figure S2 Entropy analysis of chromosome 2, containing the copy number heatmap (on the right) and the entropy signal (left). The threshold for determining aberrant regions is displayed in the entropy plot as a red line, and it is defined by the quantile 0.05 of the bootstrap distribution of entropy. Only tumor samples are included. The assignments of the regions is the same on the Table 1 of the manuscript and peaks that don't have any regions assigned represent normal CNV or low-entropy regions in normal samples.
(0.34 MB TIF)

Figure S3 Entropy analysis of chromosome 3, containing the copy number heatmap (on the right) and the entropy signal (left). The threshold for determining aberrant regions is displayed in the entropy plot as a red line, and it is defined by the quantile 0.05 of the bootstrap distribution of entropy. Only tumor samples are included. The assignments of the regions is the same on the Table 1 of the manuscript and peaks that don't have any regions assigned represent normal CNV or low-entropy regions in normal samples.
(0.33 MB TIF)

Figure S4 Entropy analysis of chromosome 4, containing the copy number heatmap (on the right) and the entropy signal (left). The threshold for determining aberrant regions is displayed in the entropy plot as a red line, and it is defined by the quantile 0.05 of the bootstrap distribution of entropy. Only tumor samples are included. The assignments of the regions is the same on the Table 1 of the manuscript and peaks that don't have any regions assigned represent normal CNV or low-entropy regions in normal samples.
(0.34 MB TIF)

Figure S5 Entropy analysis of chromosome 5, containing the copy number heatmap (on the right) and the entropy signal (left). The threshold for determining aberrant regions is displayed in the entropy plot as a red line, and it is defined by the quantile 0.05 of the bootstrap distribution of entropy. Only tumor samples are included. The assignments of the regions is the same on the Table 1 of the manuscript and peaks that don't have any regions assigned represent normal CNV or low-entropy regions in normal samples.
(0.32 MB TIF)

Figure S6 Entropy analysis of chromosome 6, containing the copy number heatmap (on the right) and the entropy signal (left). The threshold for determining aberrant regions is displayed in the entropy plot as a red line, and it is defined by the quantile 0.05 of the bootstrap distribution of entropy. Only tumor samples are included. The assignments of the regions is the same on the Table 1 of the manuscript and peaks that don't have any regions assigned represent normal CNV or low-entropy regions in normal samples.
(0.37 MB TIF)

Figure S7 Entropy analysis of chromosome 7, containing the copy number heatmap (on the right) and the entropy signal (left). The threshold for determining aberrant regions is displayed in the entropy plot as a red line, and it is defined by the quantile 0.05 of the bootstrap distribution of entropy. Only tumor samples are included. The assignments of the regions is the same on the Table 1 of the manuscript and peaks that don't have any regions assigned represent normal CNV or low-entropy regions in normal samples.
(0.48 MB TIF)

Figure S8 Entropy analysis of chromosome 8, containing the copy number heatmap (on the right) and the entropy signal (left). The threshold for determining aberrant regions is displayed in the entropy plot as a red line, and it is defined by the quantile 0.05 of the bootstrap distribution of entropy. Only tumor samples are included. The assignments of the regions is the same on the Table 1 of the manuscript and peaks that don't have any regions assigned represent normal CNV or low-entropy regions in normal samples.
(0.34 MB TIF)

Figure S9 Entropy analysis of chromosome 9, containing the copy number heatmap (on the right) and the entropy signal (left). The threshold for determining aberrant regions is displayed in the entropy plot as a red line, and it is defined by the quantile 0.05 of the bootstrap distribution of entropy. Only tumor samples are included. The assignments of the regions is the same on the Table 1 of the manuscript and peaks that don't have any regions assigned represent normal CNV or low-entropy regions in normal samples.
(0.35 MB TIF)

Figure S10 Entropy analysis of chromosome 10, containing the copy number heatmap (on the right) and the entropy signal (left). The threshold for determining aberrant regions is displayed in the entropy plot as a red line, and it is defined by the quantile 0.05 of the bootstrap distribution of entropy. Only tumor samples are included. The assignments of the regions is the same on the Table 1 of the manuscript and peaks that don't have any regions assigned represent normal CNV or low-entropy regions in normal samples.
(0.40 MB TIF)

Figure S11 Entropy analysis of chromosome 11, containing the copy number heatmap (on the right) and the entropy signal (left). The threshold for determining aberrant regions is displayed in the entropy plot as a red line, and it is defined by the quantile 0.05 of the bootstrap distribution of entropy. Only tumor samples are included. The assignments of the regions is the same on the Table 1 of the manuscript and peaks that don't have any regions assigned represent normal CNV or low-entropy regions in normal samples.
(0.39 MB TIF)

Figure S12 Entropy analysis of chromosome 12, containing the copy number heatmap (on the right) and the entropy signal (left). The threshold for determining aberrant regions is displayed in the entropy plot as a red line, and it is defined by the quantile 0.05 of the bootstrap distribution of entropy. Only tumor samples are included. The assignments of the regions is the same on the Table 1 of the manuscript and peaks that don't have any regions assigned represent normal CNV or low-entropy regions in normal samples.
(0.39 MB TIF)

Figure S13 Entropy analysis of chromosome 13, containing the copy number heatmap (on the right) and the entropy signal (left). The threshold for determining aberrant regions is displayed in the entropy plot as a red line, and it is defined by the quantile 0.05 of the bootstrap distribution of entropy. Only tumor samples are included. The assignments of the regions is the same on the Table 1 of the manuscript and peaks that don't have any regions assigned represent normal CNV or low-entropy regions in normal samples.
(0.38 MB TIF)

Figure S14 Entropy analysis of chromosome 14, containing the copy number heatmap (on the right) and the entropy signal (left). The threshold for determining aberrant regions is displayed in the entropy plot as a red line, and it is defined by the quantile 0.05 of the bootstrap distribution of entropy. Only tumor samples are included. The assignments of the regions is the same on the Table 1 of the manuscript and peaks that don't have any regions assigned represent normal CNV or low-entropy regions in normal samples.
(0.39 MB TIF)

Figure S15 Entropy analysis of chromosome 15, containing the copy number heatmap (on the right) and the entropy signal (left). The threshold for determining aberrant regions is displayed in the entropy plot as a red line, and it is defined by the quantile 0.05 of the bootstrap distribution of entropy. Only tumor samples are included. The assignments of the regions is the same on the Table 1 of the manuscript and peaks that don't have any regions assigned represent normal CNV or low-entropy regions in normal samples.
(0.34 MB TIF)

Figure S16 Entropy analysis of chromosome 16, containing the copy number heatmap (on the right) and the entropy signal (left). The threshold for determining aberrant regions is displayed in the entropy plot as a red line, and it is defined by the quantile 0.05 of the bootstrap distribution of entropy. Only tumor samples are included. The assignments of the regions is the same on the Table 1 of the manuscript and peaks that don't have any regions assigned represent normal CNV or low-entropy regions in normal samples.
(0.32 MB TIF)

Figure S17 Entropy analysis of chromosome 17, containing the copy number heatmap (on the right) and the entropy signal (left). The threshold for determining aberrant regions is displayed in the entropy plot as a red line, and it is defined by the quantile 0.05 of the bootstrap distribution of entropy. Only tumor samples are included. The assignments of the regions is the same on the Table 1 of the manuscript and peaks that don't have any regions assigned represent normal CNV or low-entropy regions in normal samples.
(0.41 MB TIF)

Figure S18 Entropy analysis of chromosome 18, containing the copy number heatmap (on the right) and the entropy signal (left). The threshold for determining aberrant regions is displayed in the entropy plot as a red line, and it is defined by the quantile 0.05 of the bootstrap distribution of entropy. Only tumor samples are included. The assignments of the regions is the same on the Table 1 of the manuscript and peaks that don't have any regions assigned represent normal CNV or low-entropy regions in normal samples.
(0.39 MB TIF)

Figure S19 Entropy analysis of chromosome 19, containing the copy number heatmap (on the right) and the entropy signal (left). The threshold for determining aberrant regions is displayed in the entropy plot as a red line, and it is defined by the quantile 0.05 of the bootstrap distribution of entropy. Only tumor samples are included. The assignments of the regions is the same on the Table 1 of the manuscript and peaks that don't have any regions assigned represent normal CNV or low-entropy regions in normal samples.
(0.38 MB TIF)

Figure S20 Entropy analysis of chromosome 20, containing the copy number heatmap (on the right) and the entropy signal (left). The threshold for determining aberrant regions is displayed in the entropy plot as a red line, and it is defined by the quantile 0.05 of the bootstrap distribution of entropy. Only tumor samples are included. The assignments of the regions is the same on the Table 1 of the manuscript and peaks that don't have any regions assigned represent normal CNV or low-entropy regions in normal samples.
(0.42 MB TIF)

Figure S21 Entropy analysis of chromosome 21, containing the copy number heatmap (on the right) and the entropy signal (left). The threshold for determining aberrant regions is displayed in the entropy plot as a red line, and it is defined by the quantile 0.05 of the bootstrap distribution of entropy. Only tumor samples are included. The assignments of the regions is the same on the Table 1 of the manuscript and peaks that don't have any regions assigned represent normal CNV or low-entropy regions in normal samples.
(0.40 MB TIF)

Figure S22 Entropy analysis of chromosome 22, containing the copy number heatmap (on the right) and the entropy signal (left). The threshold for determining aberrant regions is displayed in the entropy plot as a red line, and it is defined by the quantile 0.05 of the bootstrap distribution of entropy. Only tumor samples are included. The assignments of the regions is the same on the Table 1 of the manuscript and peaks that don't have any regions assigned represent normal CNV or low-entropy regions in normal samples.
(0.39 MB TIF)

Table S1 Low-entropy regions, including the normal CNV regions.
(0.10 MB XLS)

Table S2 Genes within low-entropy regions
(0.05 MB XLS)

Table S3 Low-entropy regions in the 60 normal samples.
(0.22 MB XLS)

Table S4 List of the samples used and the tissues where it came from. A sample code is composed by two parts, the patient code and the tissue code. For instance, the sample TCGA-02-0001-01A can be devided in tow parts: TCGA-02-0001 (patient code) and -01A (tissue coide). Therefor, the samples TCGA-02-0001-01A and TCGA-02-0001-10A came from the same patient. For further information on the sample barcode, please refer the TCGA data description (http://tcga-data.nci.nih.gov/docs/TCGA_Data_Primer.pdf).
(0.03 MB XLS)
