# Supplementary figures and images for: Exploratory Analysis of the Copy Number Alterations in Glioblastoma Multiforme
Source: PLoS One. 2008 Dec 30;3(12):e4076. doi: 10.1371/journal.pone.0004076 (PMC2605252; doi:10.1371/journal.pone.0004076)

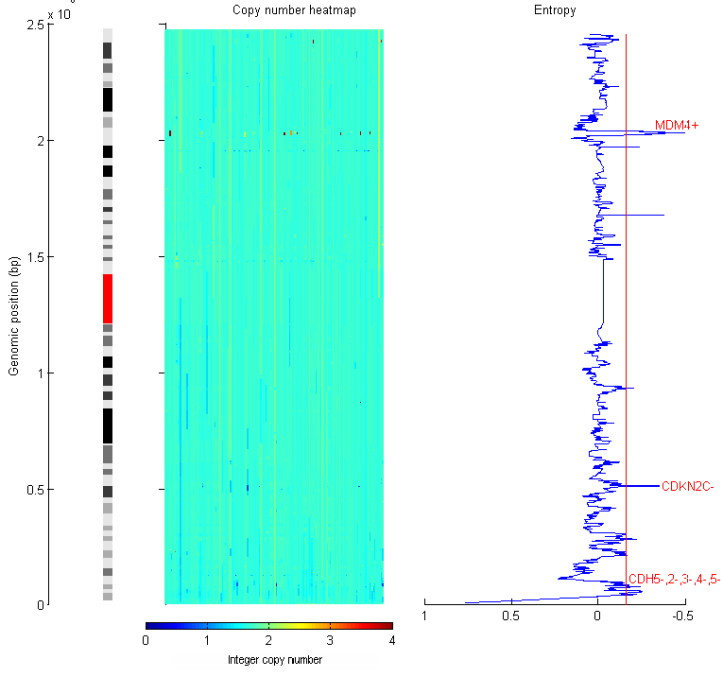

Supplement: Figure S1 — Entropy analysis of chromosome 1, containing the copy number heatmap (on the right) and the entropy signal (left). The threshold for determining aberrant regions is displayed in the entropy plot as a red line, and it is defined by the quantile 0.05 of the bootstrap distribution of entropy. Only tumor samples are included. The assignments of the regions is the same on the Table 1 of the manuscript and peaks that don't have any regions assigned represent normal CNV or low-entropy regions in normal samples. (0.26 MB TIF) [file pone.0004076.s001.tif]

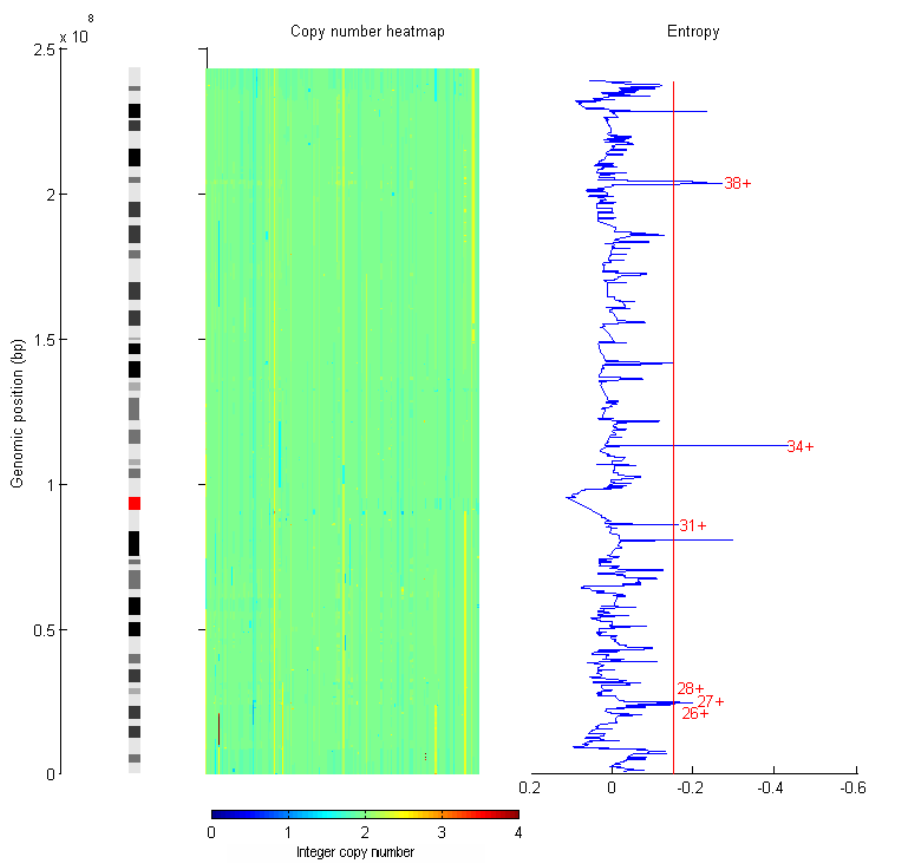

Supplement: Figure S2 — Entropy analysis of chromosome 2, containing the copy number heatmap (on the right) and the entropy signal (left). The threshold for determining aberrant regions is displayed in the entropy plot as a red line, and it is defined by the quantile 0.05 of the bootstrap distribution of entropy. Only tumor samples are included. The assignments of the regions is the same on the Table 1 of the manuscript and peaks that don't have any regions assigned represent normal CNV or low-entropy regions in normal samples. (0.34 MB TIF) [file pone.0004076.s002.tif]

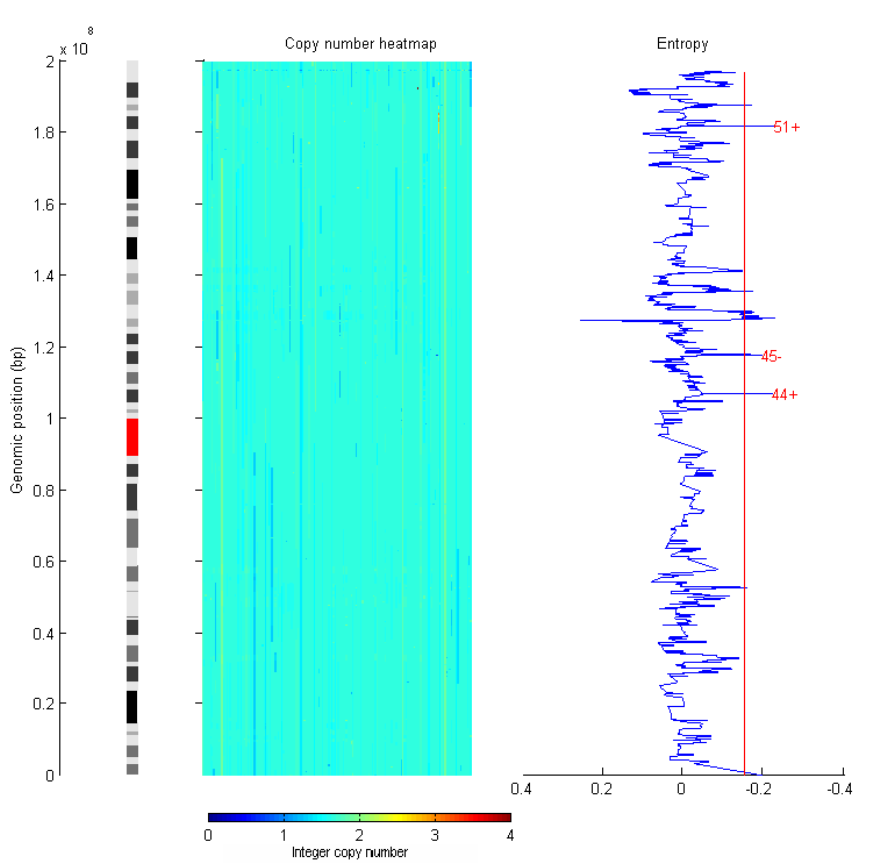

Supplement: Figure S3 — Entropy analysis of chromosome 3, containing the copy number heatmap (on the right) and the entropy signal (left). The threshold for determining aberrant regions is displayed in the entropy plot as a red line, and it is defined by the quantile 0.05 of the bootstrap distribution of entropy. Only tumor samples are included. The assignments of the regions is the same on the Table 1 of the manuscript and peaks that don't have any regions assigned represent normal CNV or low-entropy regions in normal samples. (0.33 MB TIF) [file pone.0004076.s003.tif]

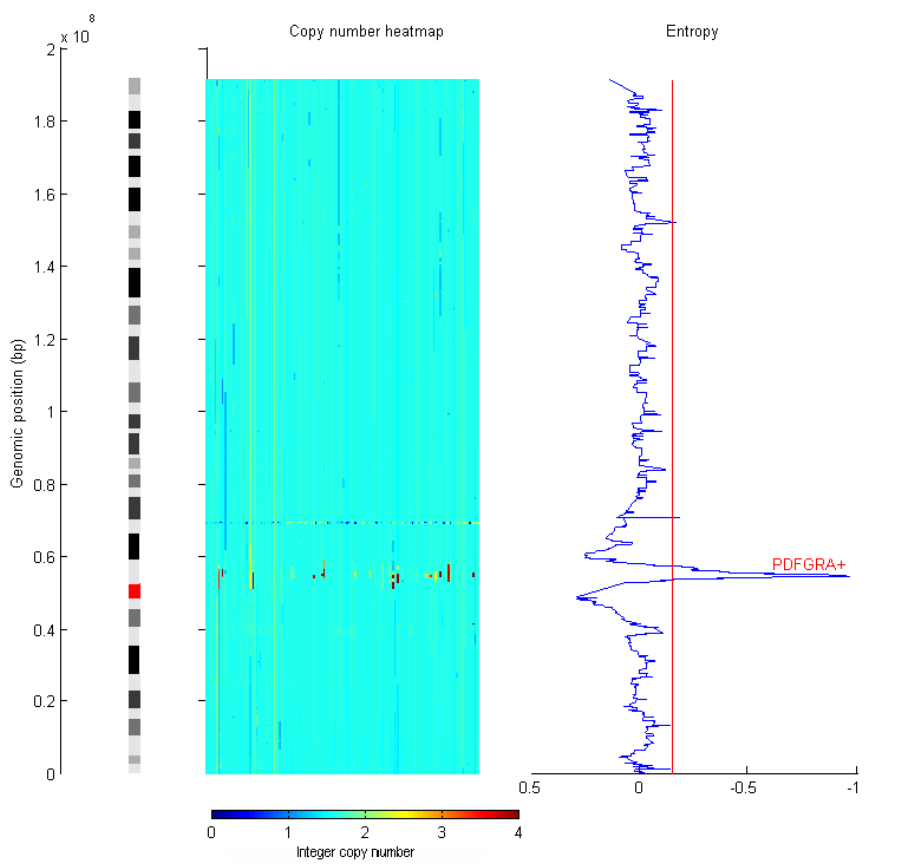

Supplement: Figure S4 — Entropy analysis of chromosome 4, containing the copy number heatmap (on the right) and the entropy signal (left). The threshold for determining aberrant regions is displayed in the entropy plot as a red line, and it is defined by the quantile 0.05 of the bootstrap distribution of entropy. Only tumor samples are included. The assignments of the regions is the same on the Table 1 of the manuscript and peaks that don't have any regions assigned represent normal CNV or low-entropy regions in normal samples. (0.34 MB TIF) [file pone.0004076.s004.tif]

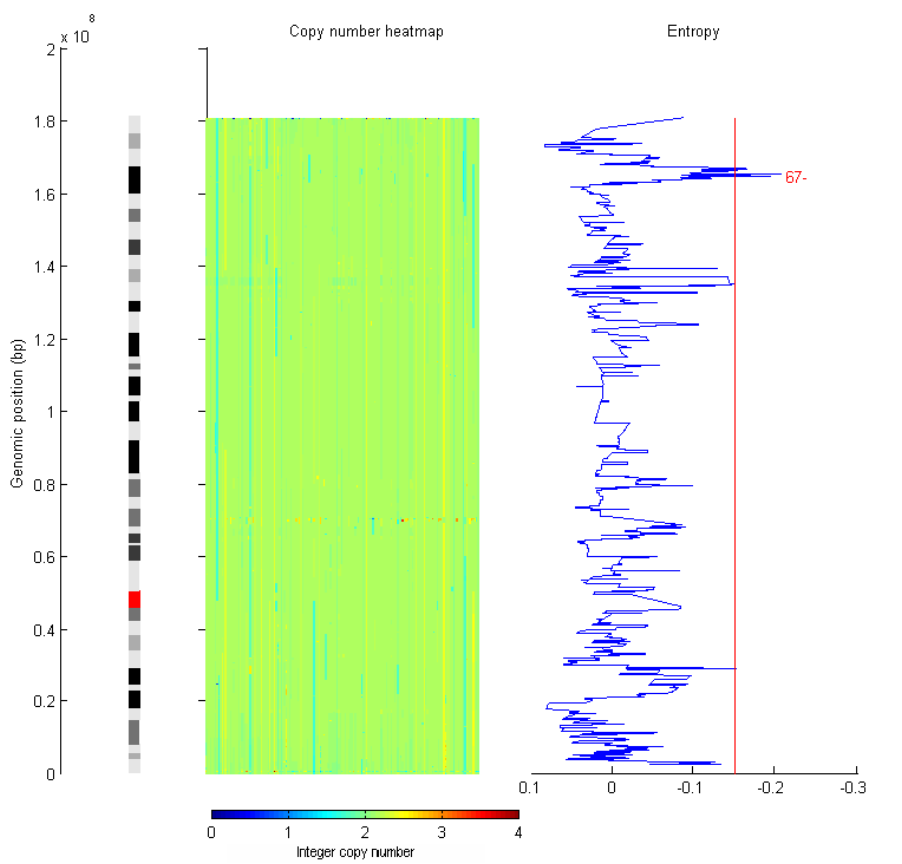

Supplement: Figure S5 — Entropy analysis of chromosome 5, containing the copy number heatmap (on the right) and the entropy signal (left). The threshold for determining aberrant regions is displayed in the entropy plot as a red line, and it is defined by the quantile 0.05 of the bootstrap distribution of entropy. Only tumor samples are included. The assignments of the regions is the same on the Table 1 of the manuscript and peaks that don't have any regions assigned represent normal CNV or low-entropy regions in normal samples. (0.32 MB TIF) [file pone.0004076.s005.tif]

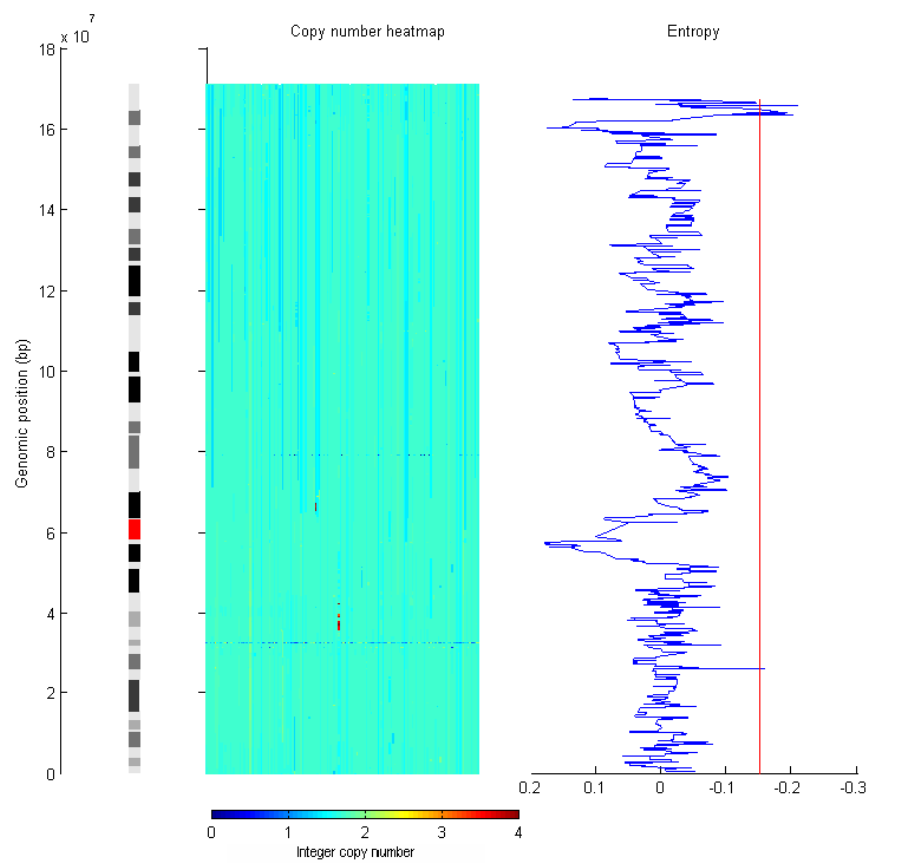

Supplement: Figure S6 — Entropy analysis of chromosome 6, containing the copy number heatmap (on the right) and the entropy signal (left). The threshold for determining aberrant regions is displayed in the entropy plot as a red line, and it is defined by the quantile 0.05 of the bootstrap distribution of entropy. Only tumor samples are included. The assignments of the regions is the same on the Table 1 of the manuscript and peaks that don't have any regions assigned represent normal CNV or low-entropy regions in normal samples. (0.37 MB TIF) [file pone.0004076.s006.tif]

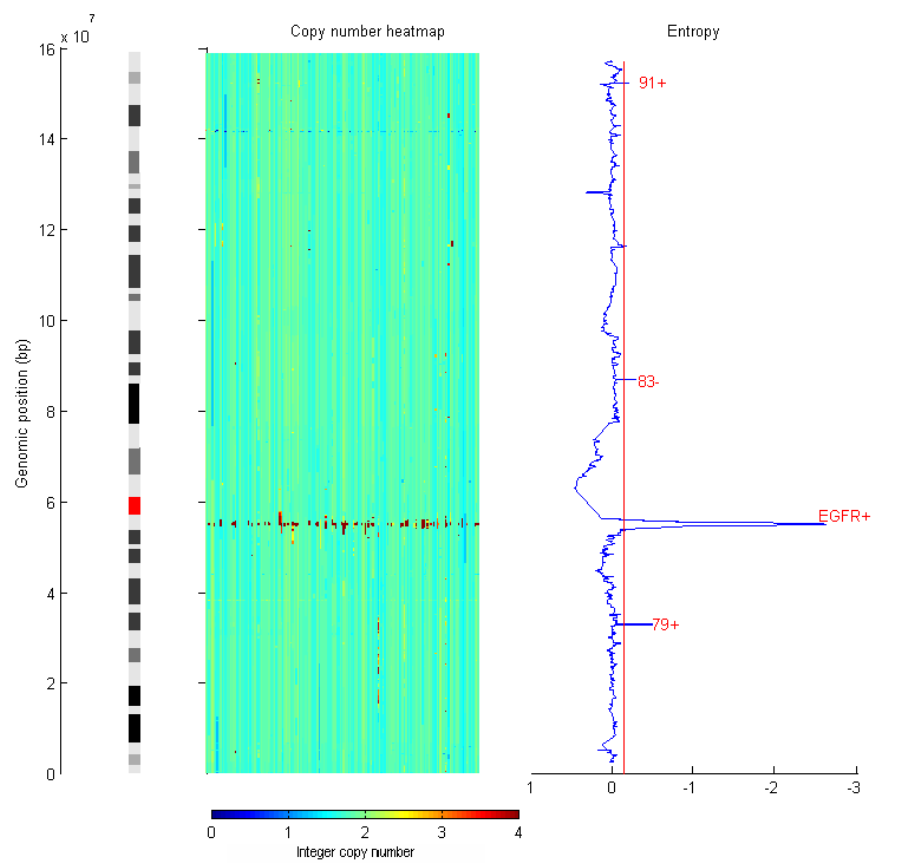

Supplement: Figure S7 — Entropy analysis of chromosome 7, containing the copy number heatmap (on the right) and the entropy signal (left). The threshold for determining aberrant regions is displayed in the entropy plot as a red line, and it is defined by the quantile 0.05 of the bootstrap distribution of entropy. Only tumor samples are included. The assignments of the regions is the same on the Table 1 of the manuscript and peaks that don't have any regions assigned represent normal CNV or low-entropy regions in normal samples. (0.48 MB TIF) [file pone.0004076.s007.tif]

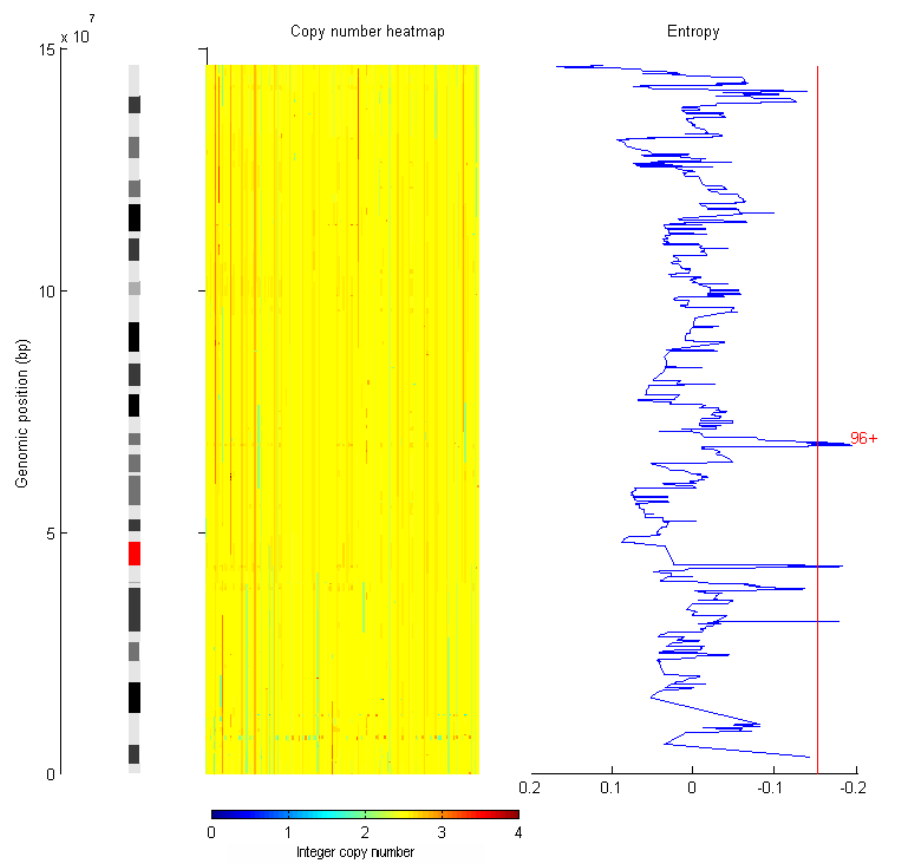

Supplement: Figure S8 — Entropy analysis of chromosome 8, containing the copy number heatmap (on the right) and the entropy signal (left). The threshold for determining aberrant regions is displayed in the entropy plot as a red line, and it is defined by the quantile 0.05 of the bootstrap distribution of entropy. Only tumor samples are included. The assignments of the regions is the same on the Table 1 of the manuscript and peaks that don't have any regions assigned represent normal CNV or low-entropy regions in normal samples. (0.34 MB TIF) [file pone.0004076.s008.tif]

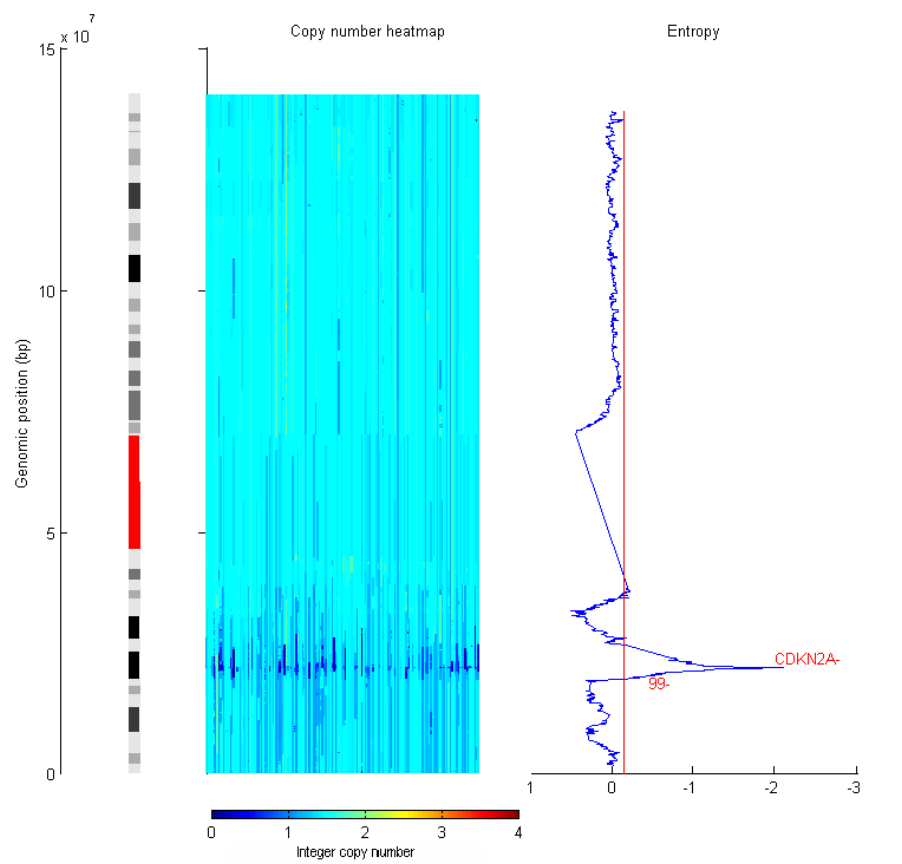

Supplement: Figure S9 — Entropy analysis of chromosome 9, containing the copy number heatmap (on the right) and the entropy signal (left). The threshold for determining aberrant regions is displayed in the entropy plot as a red line, and it is defined by the quantile 0.05 of the bootstrap distribution of entropy. Only tumor samples are included. The assignments of the regions is the same on the Table 1 of the manuscript and peaks that don't have any regions assigned represent normal CNV or low-entropy regions in normal samples. (0.35 MB TIF) [file pone.0004076.s009.tif]

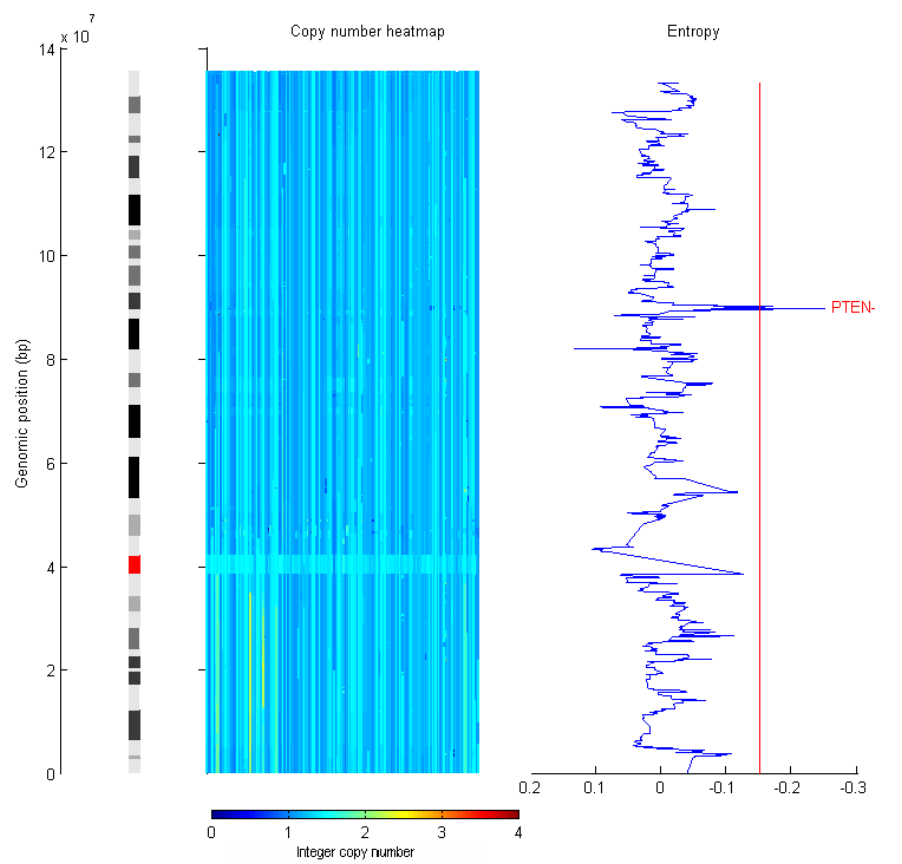

Supplement: Figure S10 — Entropy analysis of chromosome 10, containing the copy number heatmap (on the right) and the entropy signal (left). The threshold for determining aberrant regions is displayed in the entropy plot as a red line, and it is defined by the quantile 0.05 of the bootstrap distribution of entropy. Only tumor samples are included. The assignments of the regions is the same on the Table 1 of the manuscript and peaks that don't have any regions assigned represent normal CNV or low-entropy regions in normal samples. (0.40 MB TIF) [file pone.0004076.s010.tif]

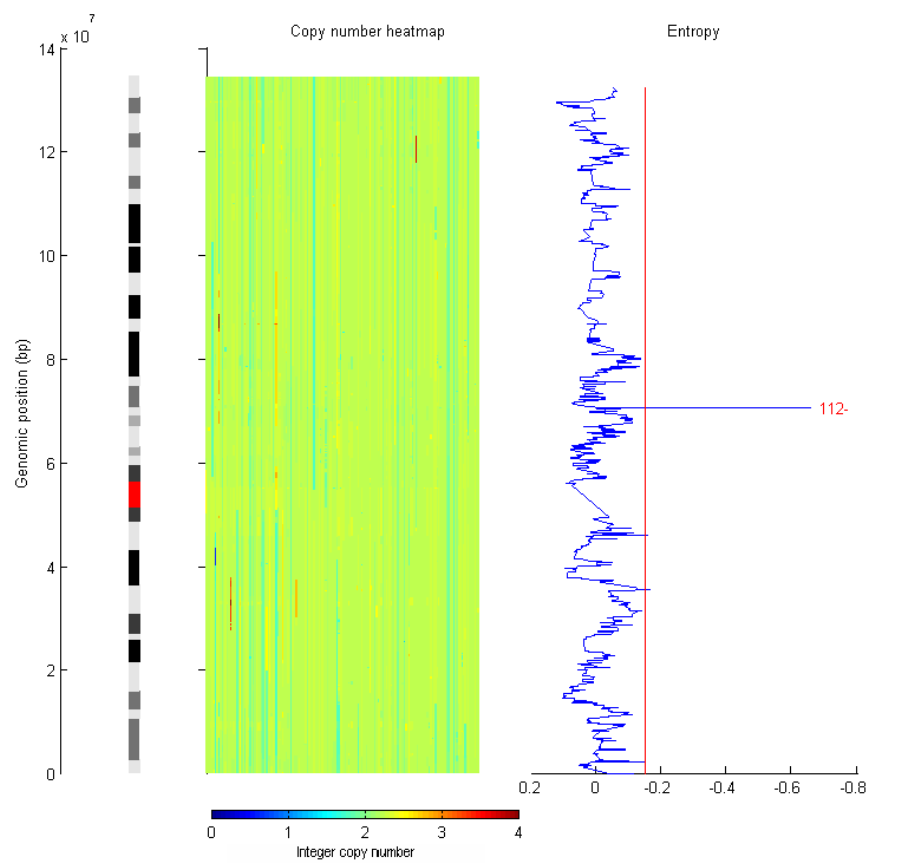

Supplement: Figure S11 — Entropy analysis of chromosome 11, containing the copy number heatmap (on the right) and the entropy signal (left). The threshold for determining aberrant regions is displayed in the entropy plot as a red line, and it is defined by the quantile 0.05 of the bootstrap distribution of entropy. Only tumor samples are included. The assignments of the regions is the same on the Table 1 of the manuscript and peaks that don't have any regions assigned represent normal CNV or low-entropy regions in normal samples. (0.39 MB TIF) [file pone.0004076.s011.tif]

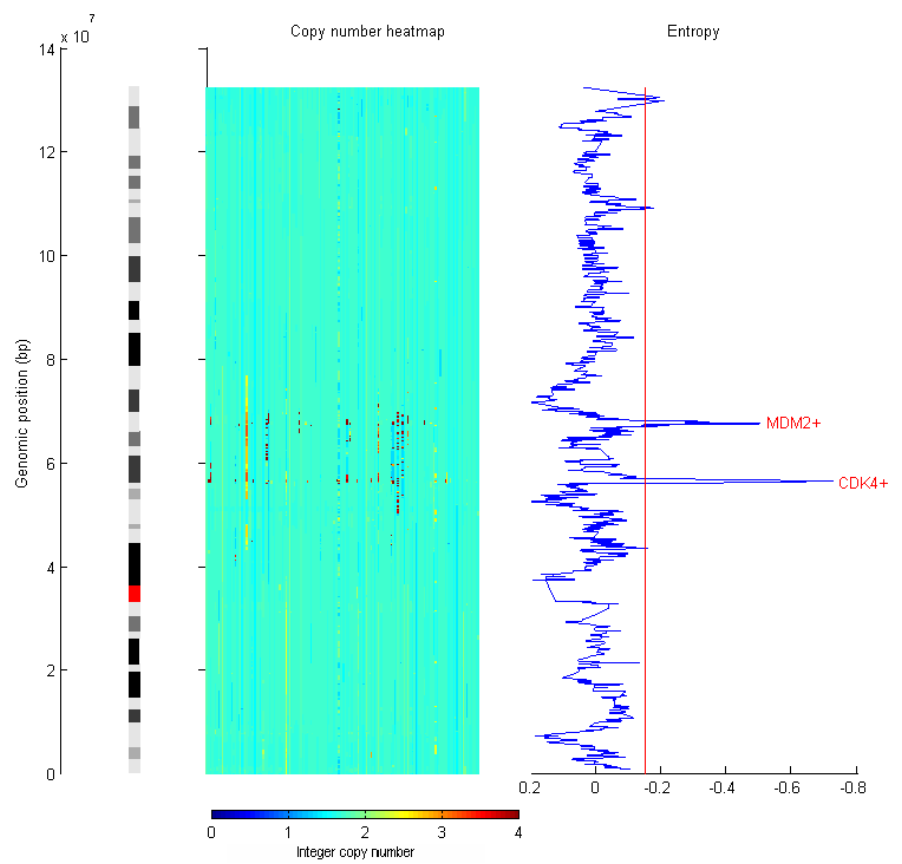

Supplement: Figure S12 — Entropy analysis of chromosome 12, containing the copy number heatmap (on the right) and the entropy signal (left). The threshold for determining aberrant regions is displayed in the entropy plot as a red line, and it is defined by the quantile 0.05 of the bootstrap distribution of entropy. Only tumor samples are included. The assignments of the regions is the same on the Table 1 of the manuscript and peaks that don't have any regions assigned represent normal CNV or low-entropy regions in normal samples. (0.39 MB TIF) [file pone.0004076.s012.tif]

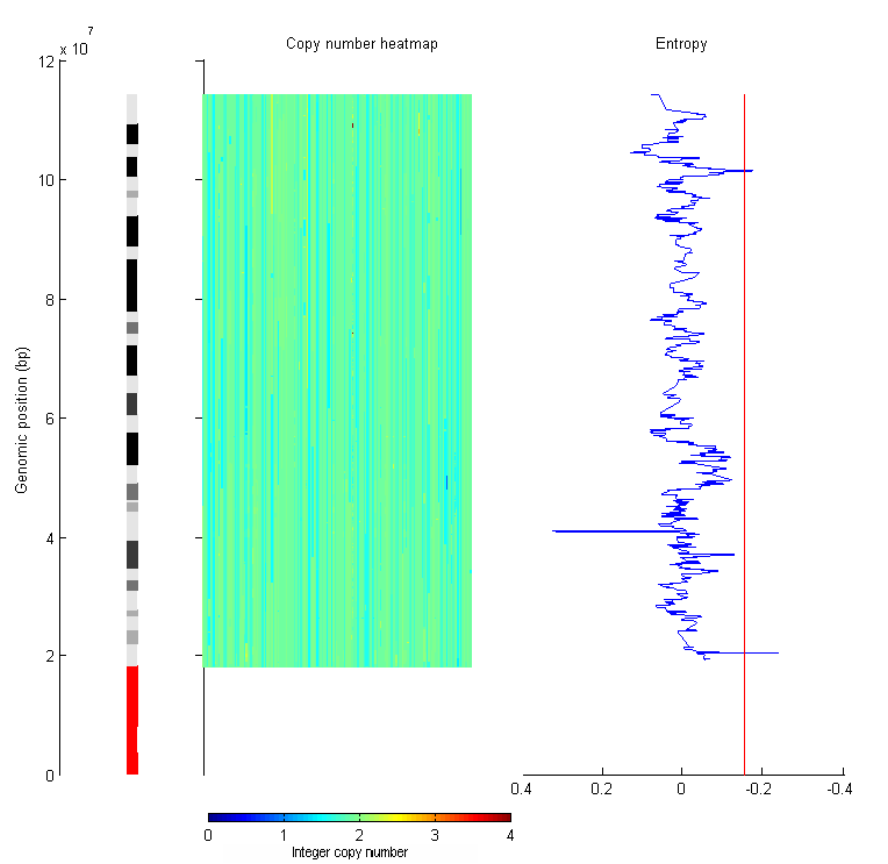

Supplement: Figure S13 — Entropy analysis of chromosome 13, containing the copy number heatmap (on the right) and the entropy signal (left). The threshold for determining aberrant regions is displayed in the entropy plot as a red line, and it is defined by the quantile 0.05 of the bootstrap distribution of entropy. Only tumor samples are included. The assignments of the regions is the same on the Table 1 of the manuscript and peaks that don't have any regions assigned represent normal CNV or low-entropy regions in normal samples. (0.38 MB TIF) [file pone.0004076.s013.tif]

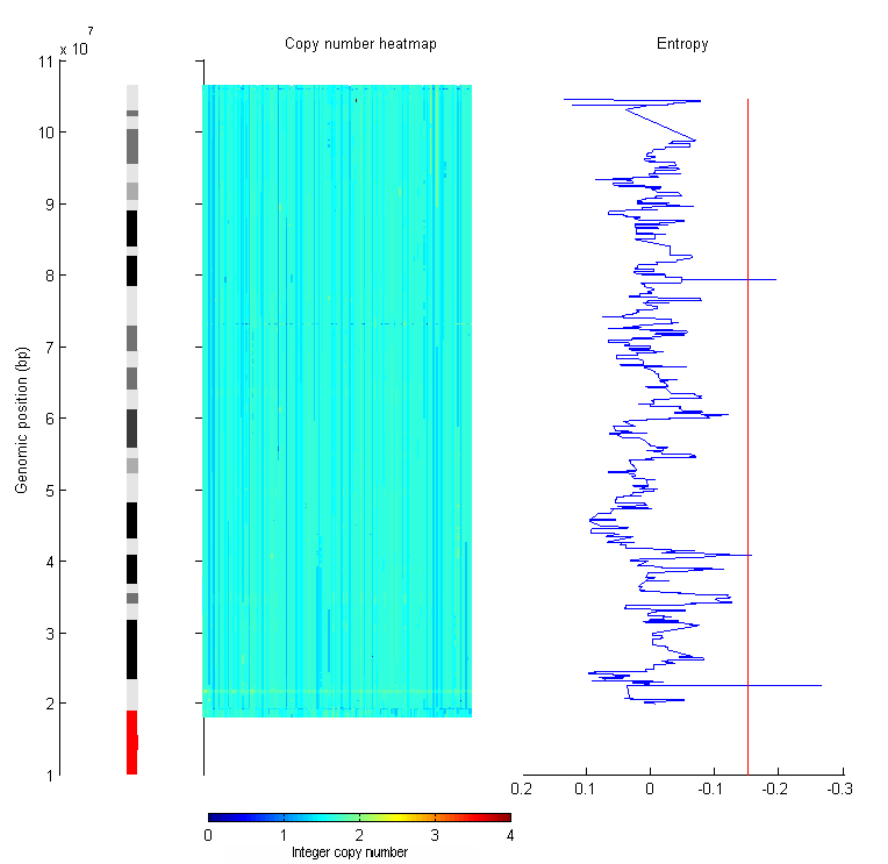

Supplement: Figure S14 — Entropy analysis of chromosome 14, containing the copy number heatmap (on the right) and the entropy signal (left). The threshold for determining aberrant regions is displayed in the entropy plot as a red line, and it is defined by the quantile 0.05 of the bootstrap distribution of entropy. Only tumor samples are included. The assignments of the regions is the same on the Table 1 of the manuscript and peaks that don't have any regions assigned represent normal CNV or low-entropy regions in normal samples. (0.39 MB TIF) [file pone.0004076.s014.tif]

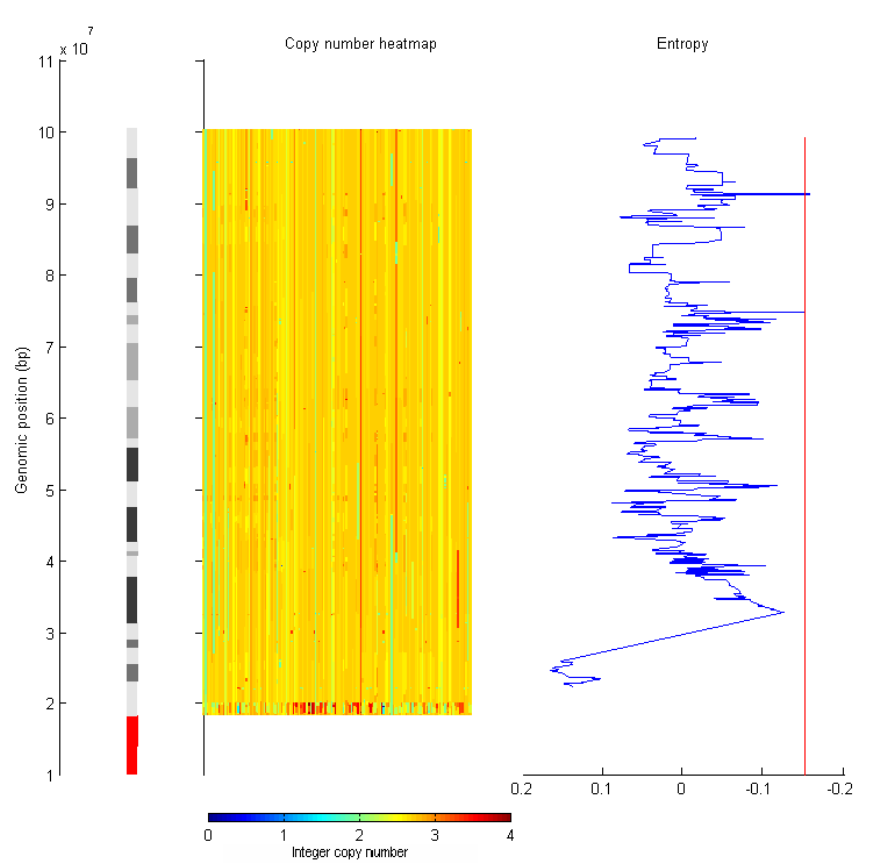

Supplement: Figure S15 — Entropy analysis of chromosome 15, containing the copy number heatmap (on the right) and the entropy signal (left). The threshold for determining aberrant regions is displayed in the entropy plot as a red line, and it is defined by the quantile 0.05 of the bootstrap distribution of entropy. Only tumor samples are included. The assignments of the regions is the same on the Table 1 of the manuscript and peaks that don't have any regions assigned represent normal CNV or low-entropy regions in normal samples. (0.34 MB TIF) [file pone.0004076.s015.tif]

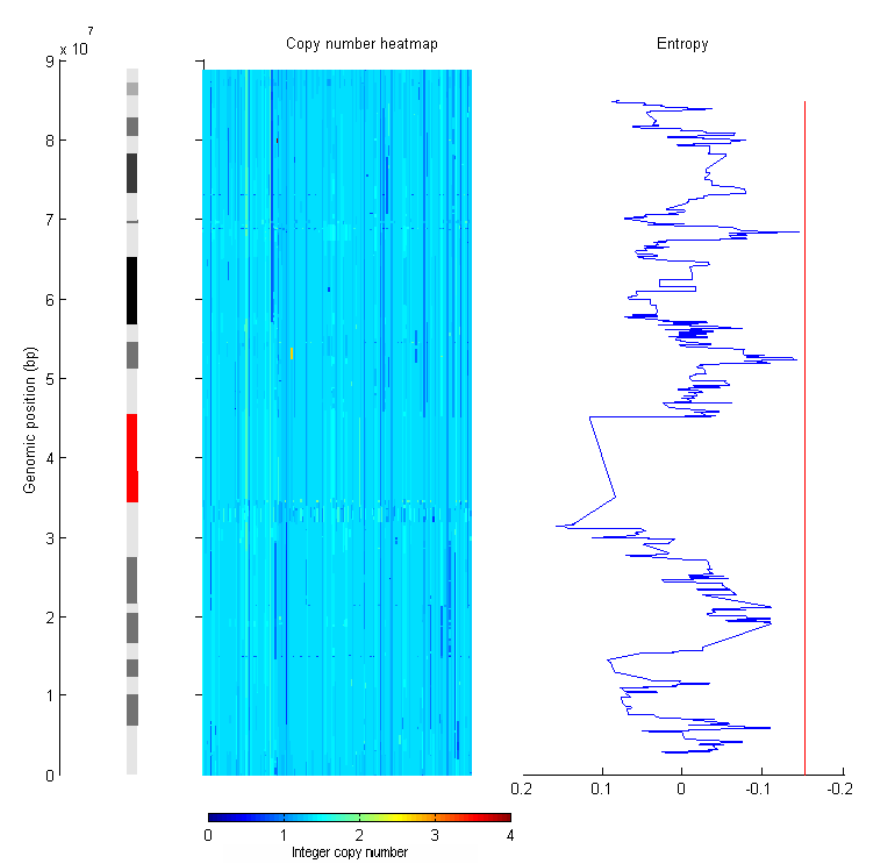

Supplement: Figure S16 — Entropy analysis of chromosome 16, containing the copy number heatmap (on the right) and the entropy signal (left). The threshold for determining aberrant regions is displayed in the entropy plot as a red line, and it is defined by the quantile 0.05 of the bootstrap distribution of entropy. Only tumor samples are included. The assignments of the regions is the same on the Table 1 of the manuscript and peaks that don't have any regions assigned represent normal CNV or low-entropy regions in normal samples. (0.32 MB TIF) [file pone.0004076.s016.tif]

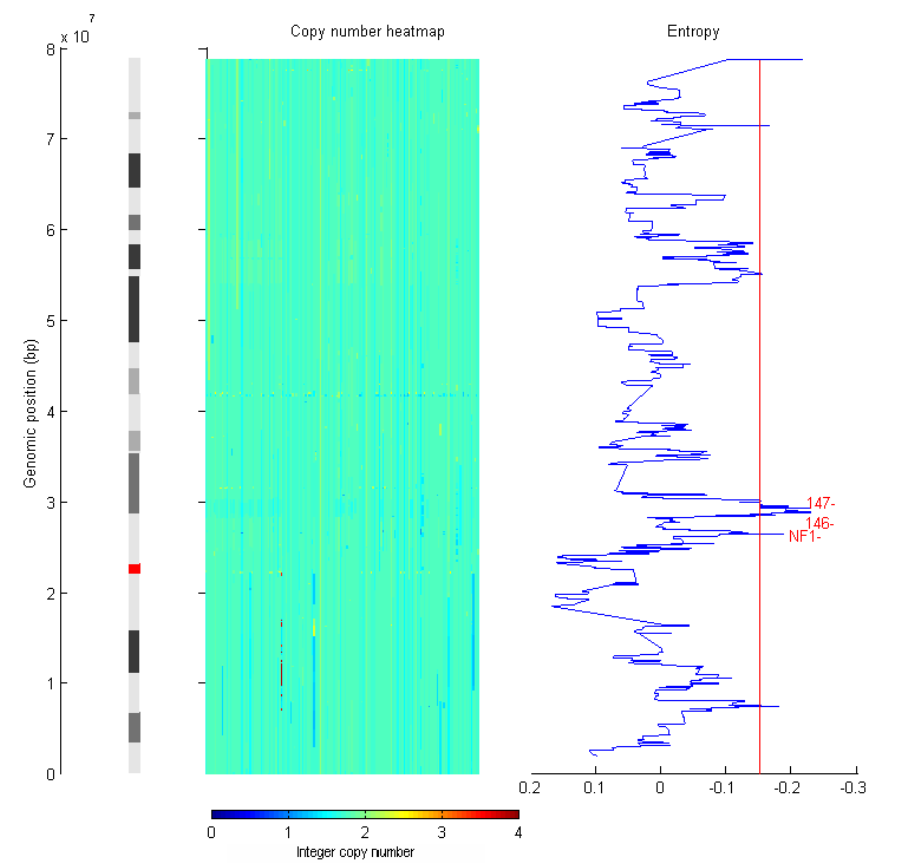

Supplement: Figure S17 — Entropy analysis of chromosome 17, containing the copy number heatmap (on the right) and the entropy signal (left). The threshold for determining aberrant regions is displayed in the entropy plot as a red line, and it is defined by the quantile 0.05 of the bootstrap distribution of entropy. Only tumor samples are included. The assignments of the regions is the same on the Table 1 of the manuscript and peaks that don't have any regions assigned represent normal CNV or low-entropy regions in normal samples. (0.41 MB TIF) [file pone.0004076.s017.tif]

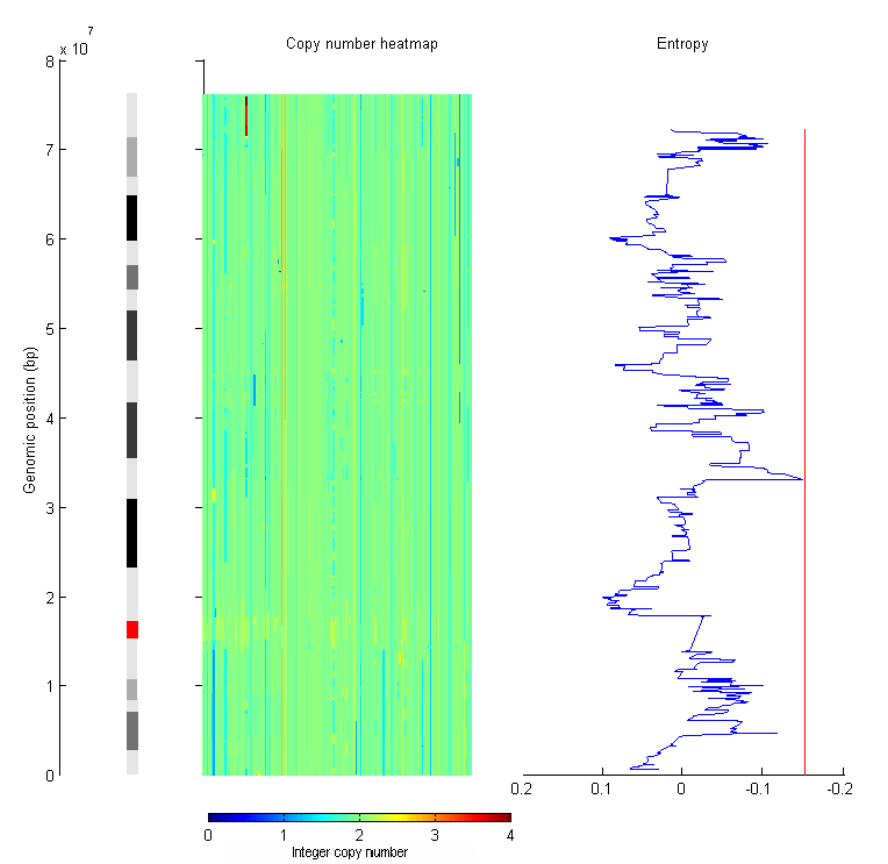

Supplement: Figure S18 — Entropy analysis of chromosome 18, containing the copy number heatmap (on the right) and the entropy signal (left). The threshold for determining aberrant regions is displayed in the entropy plot as a red line, and it is defined by the quantile 0.05 of the bootstrap distribution of entropy. Only tumor samples are included. The assignments of the regions is the same on the Table 1 of the manuscript and peaks that don't have any regions assigned represent normal CNV or low-entropy regions in normal samples. (0.39 MB TIF) [file pone.0004076.s018.tif]

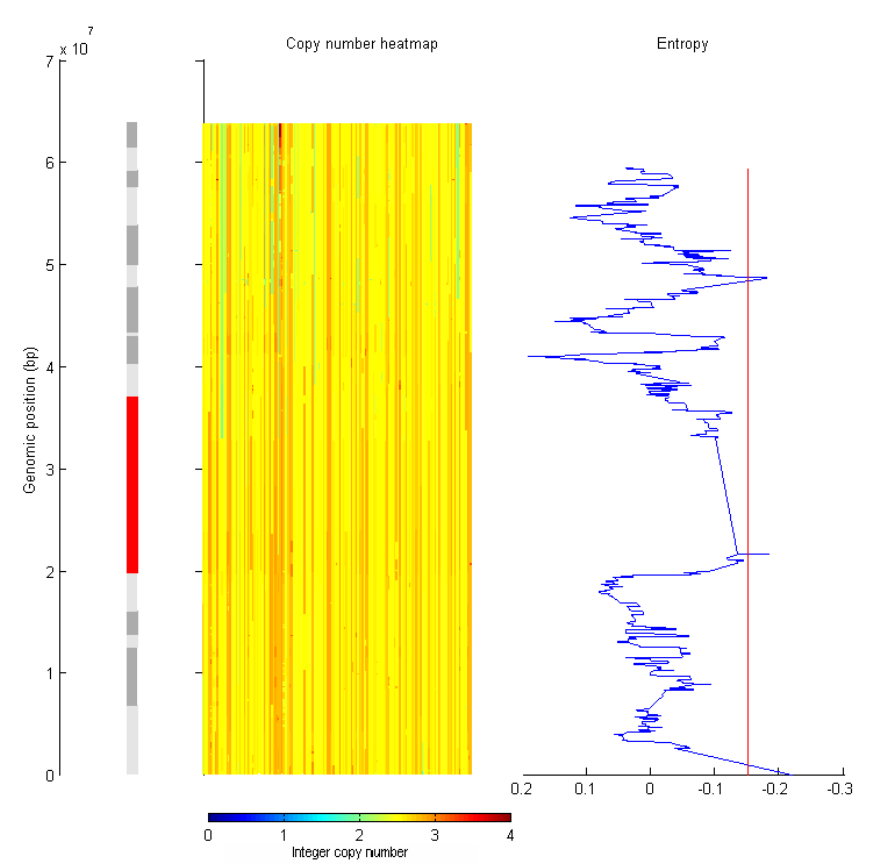

Supplement: Figure S19 — Entropy analysis of chromosome 19, containing the copy number heatmap (on the right) and the entropy signal (left). The threshold for determining aberrant regions is displayed in the entropy plot as a red line, and it is defined by the quantile 0.05 of the bootstrap distribution of entropy. Only tumor samples are included. The assignments of the regions is the same on the Table 1 of the manuscript and peaks that don't have any regions assigned represent normal CNV or low-entropy regions in normal samples. (0.38 MB TIF) [file pone.0004076.s019.tif]

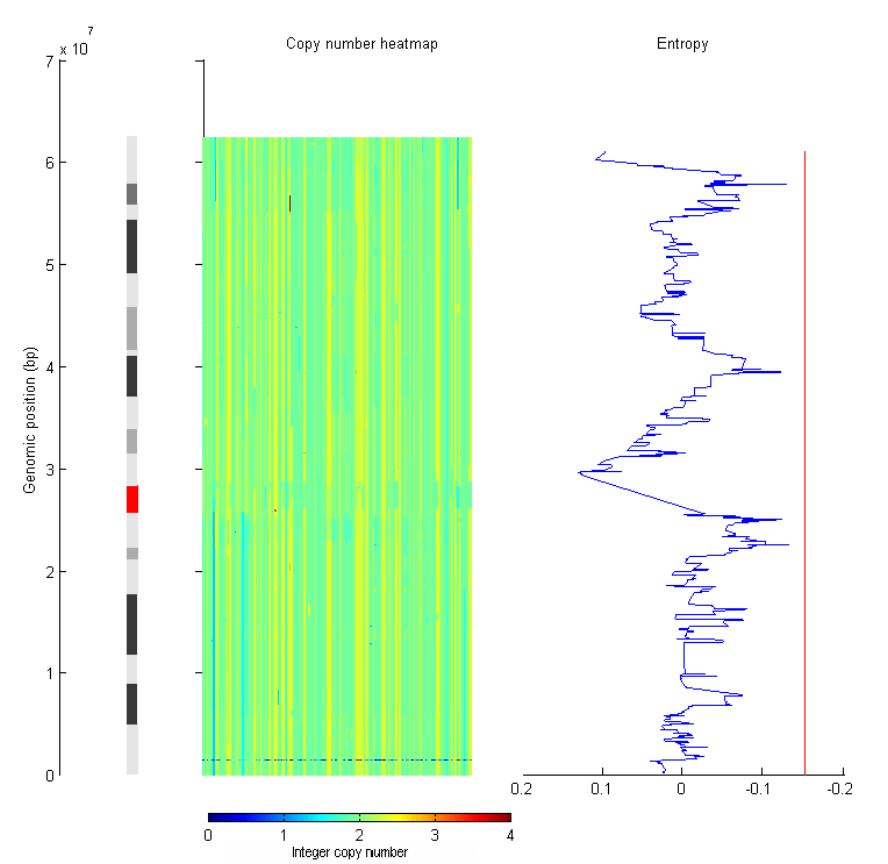

Supplement: Figure S20 — Entropy analysis of chromosome 20, containing the copy number heatmap (on the right) and the entropy signal (left). The threshold for determining aberrant regions is displayed in the entropy plot as a red line, and it is defined by the quantile 0.05 of the bootstrap distribution of entropy. Only tumor samples are included. The assignments of the regions is the same on the Table 1 of the manuscript and peaks that don't have any regions assigned represent normal CNV or low-entropy regions in normal samples. (0.42 MB TIF) [file pone.0004076.s020.tif]

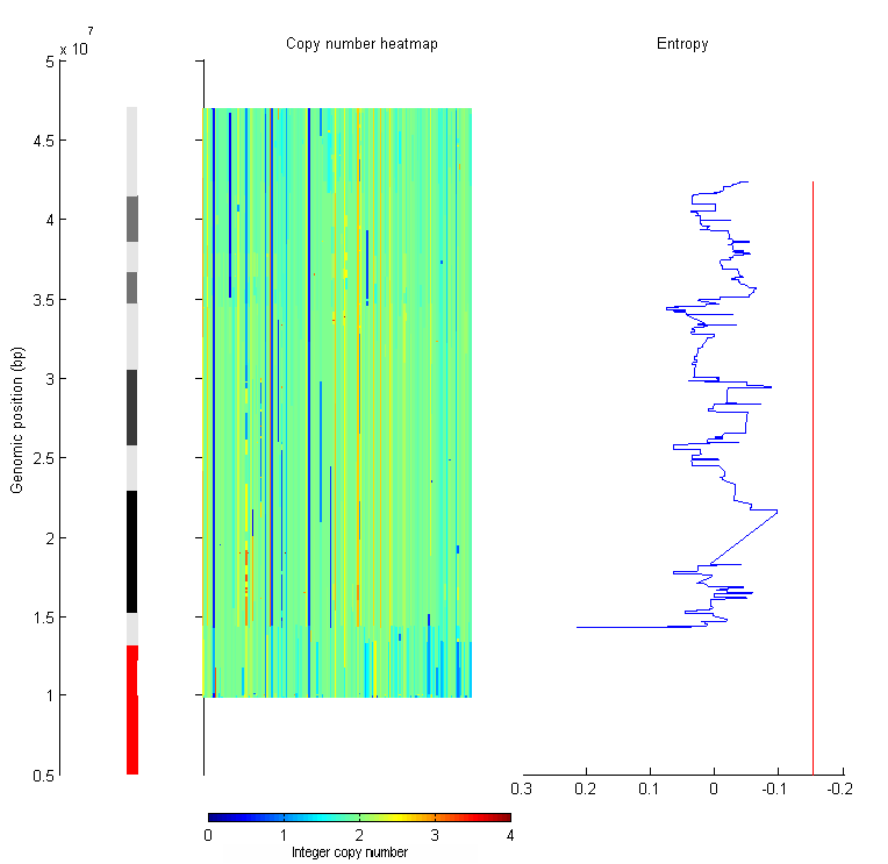

Supplement: Figure S21 — Entropy analysis of chromosome 21, containing the copy number heatmap (on the right) and the entropy signal (left). The threshold for determining aberrant regions is displayed in the entropy plot as a red line, and it is defined by the quantile 0.05 of the bootstrap distribution of entropy. Only tumor samples are included. The assignments of the regions is the same on the Table 1 of the manuscript and peaks that don't have any regions assigned represent normal CNV or low-entropy regions in normal samples. (0.40 MB TIF) [file pone.0004076.s021.tif]

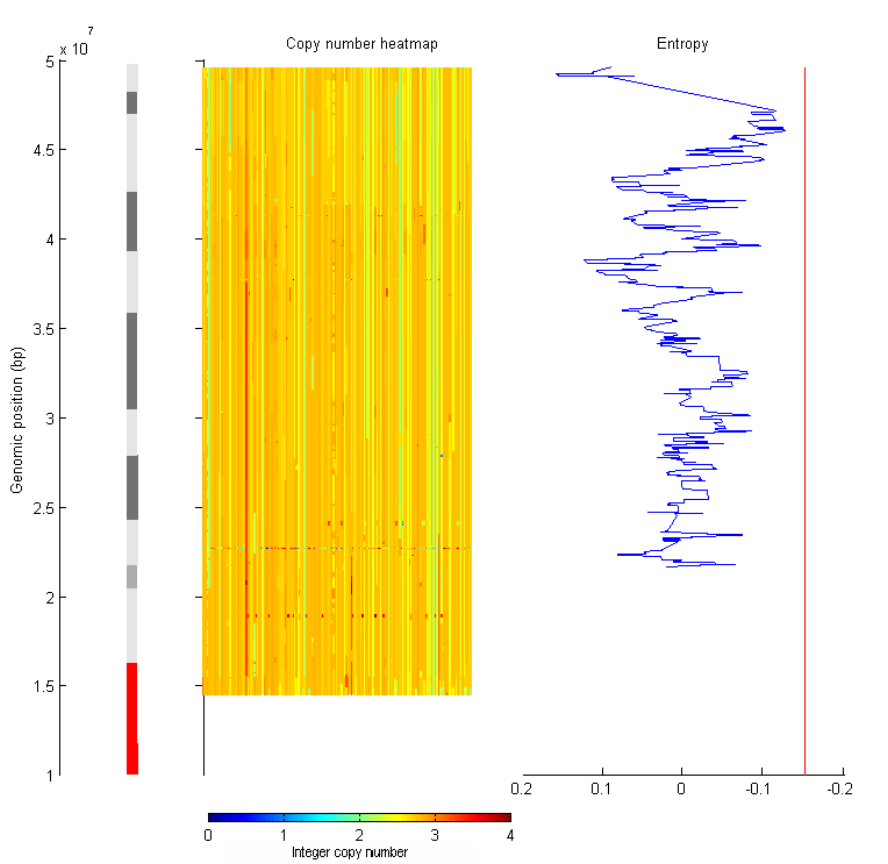

Supplement: Figure S22 — Entropy analysis of chromosome 22, containing the copy number heatmap (on the right) and the entropy signal (left). The threshold for determining aberrant regions is displayed in the entropy plot as a red line, and it is defined by the quantile 0.05 of the bootstrap distribution of entropy. Only tumor samples are included. The assignments of the regions is the same on the Table 1 of the manuscript and peaks that don't have any regions assigned represent normal CNV or low-entropy regions in normal samples. (0.39 MB TIF) [file pone.0004076.s022.tif]
